# The presence of extra chromosomes leads to genomic instability

**DOI:** 10.1038/ncomms10754

**Published:** 2016-02-15

**Authors:** Verena Passerini, Efrat Ozeri-Galai, Mirjam S. de Pagter, Neysan Donnelly, Sarah Schmalbrock, Wigard P. Kloosterman, Batsheva Kerem, Zuzana Storchová

**Affiliations:** 1 Max Planck Institute of Biochemistry, Am Klopferspitz 18, Martinsried 82152, Germany; 2 Department of Genetics, The Alexander Silberman Institute of Life Sciences, Edmond J. Safra Campus, Givat-Ram, Jerusalem 91904, Israel; 3 Department of Genetics, Center for Molecular Medicine, University Medical Center Utrecht, Universiteitsweg 100, Utrecht 3584 CG, The Netherlands; 4 Center for Integrated Protein Science Munich, Munich, Germany; 5 Technical University Kaiserslautern, Paul-Ehrlich Strasse, Building 24, Kaiserslautern 67663, Germany

## Abstract

Aneuploidy is a hallmark of cancer and underlies genetic disorders characterized by severe developmental defects, yet the molecular mechanisms explaining its effects on cellular physiology remain elusive. Here we show, using a series of human cells with defined aneuploid karyotypes, that gain of a single chromosome increases genomic instability. Next-generation sequencing and SNP-array analysis reveal accumulation of chromosomal rearrangements in aneuploids, with break point junction patterns suggestive of replication defects. Trisomic and tetrasomic cells also show increased DNA damage and sensitivity to replication stress. Strikingly, we find that aneuploidy-induced genomic instability can be explained by the reduced expression of the replicative helicase MCM2-7. Accordingly, restoring near-wild-type levels of chromatin-bound MCM helicase partly rescues the genomic instability phenotypes. Thus, gain of chromosomes triggers replication stress, thereby promoting genomic instability and possibly contributing to tumorigenesis.

## Abstract (web summary)

One of the hallmarks of cancer cells is aneuploidy, however the molecular effects are poorly understood. Here the authors show that trisomic and tetrasomic cells display increased genomic instability and reduced levels of the helicase MCM2-7.

## Graphical abstract

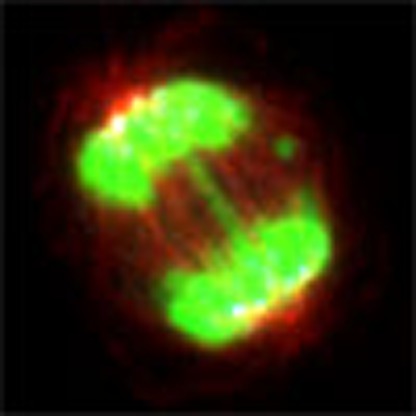

Most eukaryotic organisms are diploid as they contain two sets of chromosomes. A deviation from the normal chromosome number often markedly affects their physiology. Whole-chromosome gains or losses—called numerical aneuploidy—have particularly detrimental effects in Metazoans, as cells often suffer from impaired proliferation, increased sensitivity to proteotoxic stress and altered gene expression^1^,^2^,^3^,^4^. Structural aneuploidy, characterized by sub-chromosomal unbalanced rearrangements, that is, duplications or deletions of large genomic regions, also affects cellular functions. Both numerical and structural aneuploidy are found in nearly 75% of malignant tumours^5^, where they are often associated with ongoing whole-chromosome gains and losses, a process known as chromosomal instability (CIN), and with additional structural rearrangements due to genomic instability (GIN). Both CIN and GIN phenotypes promote cancer and contribute to tumour heterogeneity and drug resistance^6^,^7^.

Experiments in mouse models carrying mutations that impair faithful chromosome segregation revealed that the resulting CIN is sufficient to trigger tumour formation in some tissues, although it should be noted that CIN and aneuploidy also exert tumour-suppressive effects (reviewed in ref. 8). Mounting evidence shows that GIN and replication stress occur early in tumorigenesis, and many known oncogenes and oncogenic mutations act by triggering replication stress and subsequently genomic instability^9^,^10^,^11^. The occurrence of both structural and numerical aneuploidy in early stages of cancer provokes the question of whether there is a link between these two features. It was recently shown that lagging chromosomes suffer from breakage during cytokinesis^12^. Missegregated chromosomes are often contained in micronuclei in daughter cells, where they experience DNA damage likely due to aberrant replication^13^. Chromosomally unstable colorectal cancer cells show elevated DNA replication stress that was proposed to further promote chromosome missegregation^14^. Aneuploidy also correlates with chromosomal and genomic instability in transformed Chinese hamster embryo cells^15^. Yet, delineating the functional relationship between numerical aneuploidy and genomic instability, and the molecular mechanisms involved has remained challenging.

To understand the relationship between CIN and GIN, it is crucial to determine whether and how numerical aneuploidy by itself affects genome stability. Recently established model cells with defined aneuploid karyotypes have facilitated the analysis of the immediate consequences of aneuploidy *per se*^1^,^4^,^16^,^17^,^18^,^19^. Direct comparison of cognate euploid and aneuploid cells revealed that aneuploidy triggers distinct and conserved changes in gene expression^20^,^21^. Addition of even a single extra chromosome causes profound defects in the maintenance of proteostasis as aneuploid cells are more sensitive to inhibitors of protein folding, protein translation and degradation, activate protein degradation pathways and show marked protein folding defects^3^,^4^,^5^,^18^,^22^,^23^. Observations in budding and fission yeasts suggested that aneuploidy impairs the fidelity of chromosome segregation, and increases mutation and recombination rates, although the underlying mechanisms remain enigmatic^17^,^24^,^25^,^26^. Analysis of trisomy of chromosome 7 or 13 in the p53-deficient cancer cell line DLD1 revealed increased chromosome missegregation of the extra chromosome and a frequent cytokinesis failure in trisomy 13 (ref. 27). Yet, no systematic analysis of the effects of aneuploidy on the maintenance of genome stability has been performed in higher eukaryotes so far.

Here we use a series of trisomic and tetrasomic human cells with defined karyotypes derived from near-diploid and chromosomally stable parental cell lines. By direct comparison with the parental cell lines, we found that addition of even a single chromosome is associated with accumulation of replication-related DNA damage and chromosomal rearrangements. Strikingly, we found profound abundance changes of proteins involved in DNA replication in response to the presence of extra chromosomes, in particular, consistently low levels of the replicative helicase MCM2-7. Our data provide the first evidence that the presence of even a single extra chromosome in human cells triggers genomic instability by impairing DNA replication, thus establishing a new link between numerical and structural aneuploidy that might play a role in cancer.

## Results

### Accumulation of pre-mitotic DNA errors in aneuploid cells

**Figure 1:**
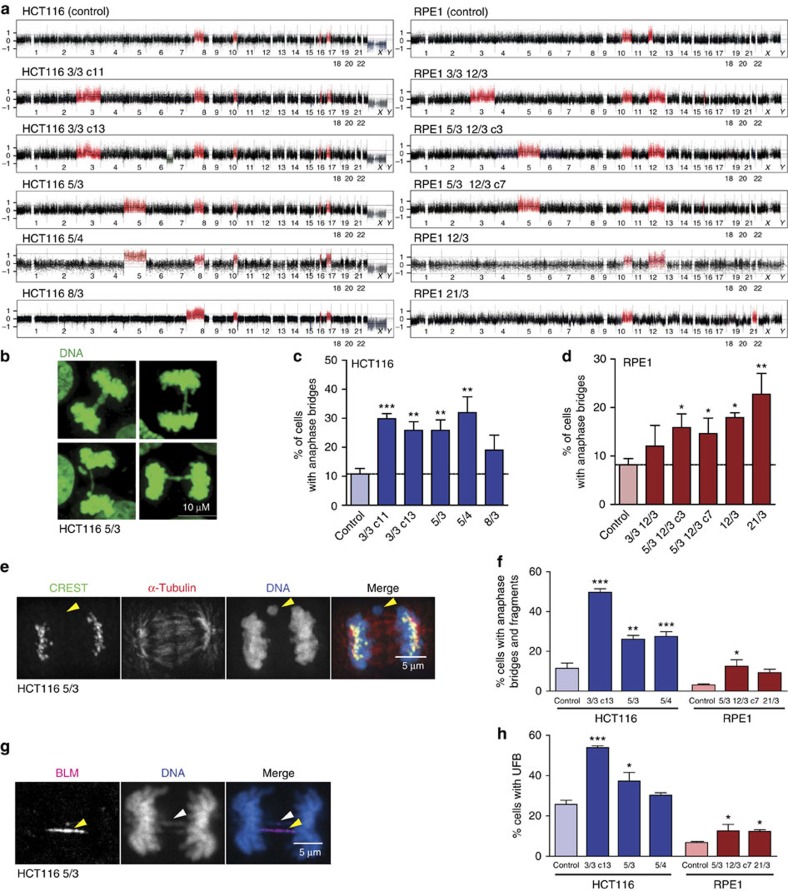
Trisomy and tetrasomy elevates the frequency of pre-mitotic errors. (**a**) Chromosome copy number changes in the parental HCT116, RPE1 and the respective trisomic and tetrasomic cell lines. Chromosome gains are marked in red and chromosome losses in grey. Note that both HCT116 and RPE1 contain several previously documented structural copy number variations that remained largely unchanged in the trisomic and tetrasomic derivatives. The identity of the extra chromosome and the number of copies were used for identification of each cell line, for example, HCT116 3/3 contain three copies of chromosome 3. Two cell lines with identical trisomies, but originating from different single cells, were selected for HCT116 3/3: clone 11 and clone 13 (c11 and c13) and for RPE 5/3 12/3 (c3 and c7) to determine the effect of clonal differences. (**b**) Representative images of a HCT116 5/3 anaphase cell with anaphase bridges. (**c**,**d**) Quantification of anaphase bridges in controls HCT116 and RPE1, and the respective trisomic and tetrasomic derivatives. (**e**) Representative images of an HCT116 5/3 anaphase cell stained with DAPI, anti-centromere antibody CREST and anti-α-tubulin. Arrowhead marks an acentric chromosome fragment. (**f**) Quantification of acentric chromosomal fragments and anaphase bridges. Bridges extend fully between DNA masses; acentric fragments were distinguished from whole-lagging chromosomes by absence of the CREST signal. (**g**) Examples of HCT116 5/3 anaphase cells stained with DAPI and antibodies against BLM (yellow arrowheads), which bind to UFBs. White arrowhead marks an anaphase bridge. (**h**) Quantification of UFBs. Plots (**c**,**d**,**f** and **h**) show mean±s.e.m. of three independent experiments. At least 100 anaphases were scored in each experiment in **c**,**d**,**f** and **h**; in RPE1 21/3, only 68 (**d**), 51 (**f**) and 54 (**h**) anaphase cells were scored in each experiment. Nonparametric *t*-test; *P<0.05, **P<0.01 and ***P<0.001.

To determine whether the presence of one or two extra chromosomes induces genomic instability, we created a panel of trisomic and tetrasomic human cell lines using micronuclei-mediated chromosome transfer (MMCT; see Supplementary Information for further details). We analysed five different cell lines that were derived from the chromosomally stable colorectal cell line HCT116 and five cell lines originating from the primary human cell line RPE1 (retinal pigment epithelium) immortalized by the overexpression of human telomerase. Each of these cell lines contained an extra copy of either chromosome 3, 5, 8, 12 or 21. The chromosomal content was verified by single-nucleotide polymorphism (SNP) array analysis, and the cell lines were named according to the identity of the extra chromosome and its copy number, for example, HCT116 8/3 for trisomy of chromosome 8 in the HCT116 cell line (Fig. 1a). Evaluation of microscopic images of anaphases revealed that all 10 analysed trisomic and tetrasomic cells contained anaphase bridges at higher frequency than the parental cell lines (Fig. 1b–d). This finding was further confirmed using high-resolution imaging combined with staining with a centromere-specific CREST antibody. On average, 46% of HCT116 3/3, 25% of HCT116 5/3 and 28% of HCT116 5/4 anaphase cells contained anaphase bridges and broken chromosome fragments devoid of centromeric staining that are indicative of pre-mitotic errors, whereas only 10% of HCT116 anaphase cells showed similar defects (Fig. 1e,f). A similar trend was observed in RPE1-derived cell lines, with an increase from 2.5% in control to 12.7% in RPE1 5/3 12/3 and 9% in RPE1 21/3 (Fig. 1f). In contrast, the analysed cell lines showed a mild, but insignificant increase in the frequency of lagging chromosomes in comparison with the parental HCT116 or RPE1 cells (Supplementary Fig. 1a–e).

Next, we analysed the occurrence of ultrafine anaphase DNA bridges (UFBs), thread-like DNA structures that associate with the Bloom's syndrome helicase (BLM) protein and link the dividing DNA masses^28^. UFBs originate either from DNA catenanes or from replication or recombination intermediates, and their occurrence is suggestive of un-replicated or abnormal DNA structures^28^. Staining with an antibody against Bloom helicase (BLM) revealed increased frequency of UFBs by up to 60% in the trisomic and tetrasomic cells compared with controls (Fig. 1g,h). Collectively, these findings suggest that the presence of even a single extra chromosome increases the frequency of pre-mitotic errors, while chromosome segregation is not significantly impaired.

### DNA damage and chromosome breaks increase with aneuploidy

**Figure 2:**
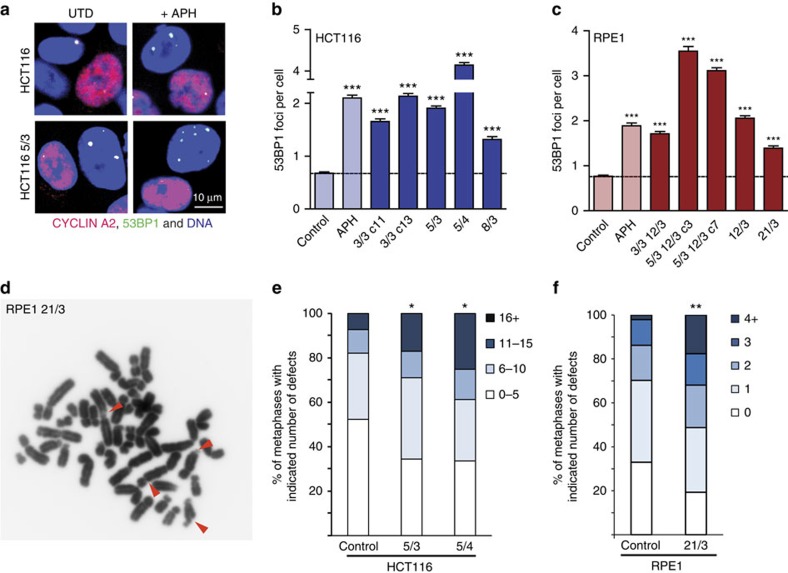
Trisomy and tetrasomy elevates DNA damage. (**a**) Example of control and aneuploid cells stained with DAPI and antibody against 53BP1 and cyclin A2 to distinguish G1 cells (cyclin A2 negative). (**b**) Average number of 53BP1 foci per G1-phase cell counted in untreated HCT116 (control), upon treatment with 0.25 μM aphidicolin (APH) and in the untreated aneuploid derivatives. (**c**) Average number of 53BP1 foci per G1-phase cell counted in untreated RPE1 (control), upon exposure to 0.25 μM aphidicolin (APH) and in the untreated aneuploid derivatives. All plots show mean±s.e.m. of three independent experiments, at least 500 cyclin A-negative cells were scored in each experiment. Nonparametric *t*-test; ***P<0.0001. (**d**) Structural aberrations, such as gaps and constrictions, on metaphase chromosomes from RPE1 21/3 cells grown under replication stress conditions (0.1 μM aphidicolin and 0.73 mM caffeine). Representative metaphase spread; aberrations are indicated by red arrowheads. (**e**) Quantification of chromosomal aberrations detected in metaphase spreads of HCT116 cells exposed to 0.3 μM aphidicolin for 24 h. Fisher's exact test, *P<0.05, *n*=94 (HCT116), 93 (5/3) and 95 (5/4) metaphases analysed in two independent experiments. (**f**) Distribution of metaphases according to the number of gaps and constrictions in RPE1, and its trisomic derivative under treatment with 0.1 μM aphidicolin and 0.73 mM caffeine for 24 h. Fisher's exact test, **P<0.01, *n*=94 (RPE1) and 119 (21/3) metaphases analysed in two independent experiments.

In order to determine whether trisomy and tetrasomy lead to increased levels of DNA damage, we determined the occurrence of 53BP1-containing nuclear bodies in cyclin A-negative G1 cells. Such 53BP1 foci have been suggested to mark unrepaired replication-induced DNA lesions that persist through mitosis^29^. We determined the number of 53BP1 foci in G1 cells using automatized image acquisition and analysis (Fig. 2a). The presence of extra chromosomes caused a significant two- to fourfold increase in the average number of 53BP1 foci per cell as well as a higher percentage of cells with >3 53BP1 foci in all trisomic and tetrasomic cell lines (Fig. 2b,c and Supplementary Fig. 2a,b). Of note, the numbers of 53BP1 foci tend to correlate with the amount of excessive DNA (Supplementary Fig. 2c). Treatment with the replication inhibitor aphidicolin further increased the number of 53BP1 foci in the tested cell lines (Supplementary Fig. 2d,e).

The higher levels of DNA damage may indicate perturbed DNA replication and/or repair at genomic regions sensitive to replication stress defined as fragile sites^30^ that can be observed as structural aberrations on metaphase chromosomes. Our analysis of metaphase spreads from cells grown under normal conditions showed a small, but insignificant trend indicative of higher fragility in response to extra chromosomes (Supplementary Fig. 2f,g). Aphidicolin treatment evoked significantly higher numbers of structural aberrations in trisomic and tetrasomic cells than in control HCT116 and RPE1 cells (Fig. 2d–f). While ∼50% of the control HCT116 cells contained >5 breaks, gaps and constrictions, a similar level of aberrations was observed in almost 70% of the metaphases in the trisomic HCT116 5/3 (Fig. 2e). In HCT116, the effect scales with aneuploidy as 25% of metaphases showed very high fragility (>16 gaps and breaks) in tetrasomic cells compared with only 7% of the control diploid cells (Fig. 2e). Increased levels of structural aberrations were also found in trisomic RPE1 21/3 cells compared with the control cells (Fig. 2f). Taken together, the addition of an extra chromosome leads to increased DNA damage and significantly higher levels of chromosome fragility under replication stress.

### Extra chromosomes increase sensitivity to replication stress

**Figure 3:**
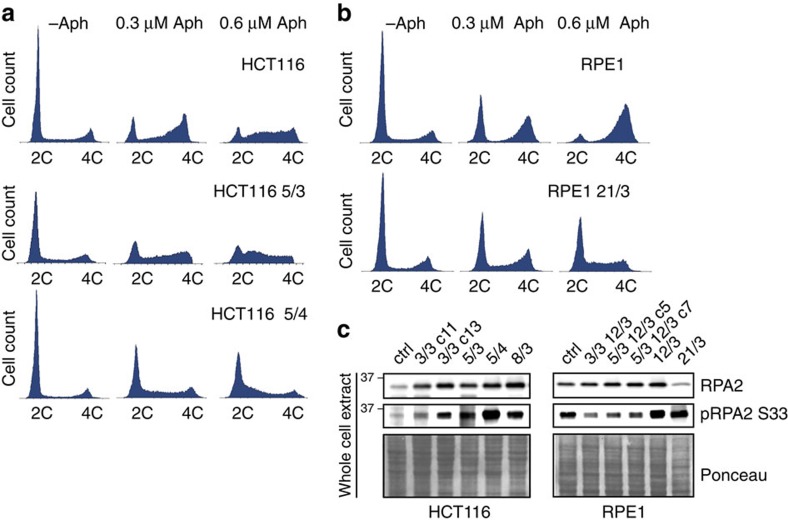
Altered replication dynamics in trisomic and tetrasomic cells. (**a**) Cell cycle profiles of HCT116, HCT116 5/3 and HCT116 5/4, and (**b**) control RPE1 and trisomic RPE1 21/3 under normal conditions, and also upon treatment with the replication inhibitor aphidicolin. (**c**) Levels of total and phosphorylated RPA2 in the parental cell lines and the trisomic and tetrasomic cell lines.

Replication stress conditions alter the cell cycle profile by slowing the progression of the cells through S-phase and/or by arresting the cells at the S-phase or G2/M checkpoints. Cell cycle analysis demonstrated that whereas under normal conditions both aneuploid HCT116 5/3 and 5/4, and their cognate controls showed nearly identical cell cycle profiles, there was a marked difference upon replication stress (Fig. 3a). Treatment with a low aphidicolin concentration led to an accumulation in late S-phase and G2/M in control cells, and this accumulation was further increased upon stronger replication stress (Fig. 3a). In contrast, trisomic HCT116 5/3 cells accumulated in S-phase following treatment with aphidicolin in a dose-dependent manner (Fig. 3a). The tetrasomic HCT116 5/4 cells were even more sensitive than trisomic cells, as a large fraction accumulated early in S-phase even in the low aphidicolin concentration (Fig. 3a), suggesting that the defects scale with the size of the extra chromosome. Similar results were found in RPE1-derived cells, in which aneuploidy caused arrest earlier in the cell cycle compared with the control cell line for each aphidicolin concentration analysed (Fig. 3b). These results suggest changes in progression through replication in cells with extra chromosomes. Quantifying the average incorporation of a thymidine analogue 5-ethynyl-2′-deoxyuridine (EdU) followed by its visualization by copper-catalyzed azide alkyne cycloaddition (Click reaction) showed that all trisomic and tetrasomic cells replicated their DNA slower than the control cells (Supplementary Fig. 3a–c). In addition, most cell lines with extra chromosome activated the replication checkpoint as we observed increased phosphorylation of RPA2 even under non-perturbed conditions (Fig. 3c). These findings are in agreement with our previous observations that the progression through S-phase is impaired by the presence of extra chromosomes^4^.

### Aneuploidy causes genomic rearrangements

**Figure 4:**
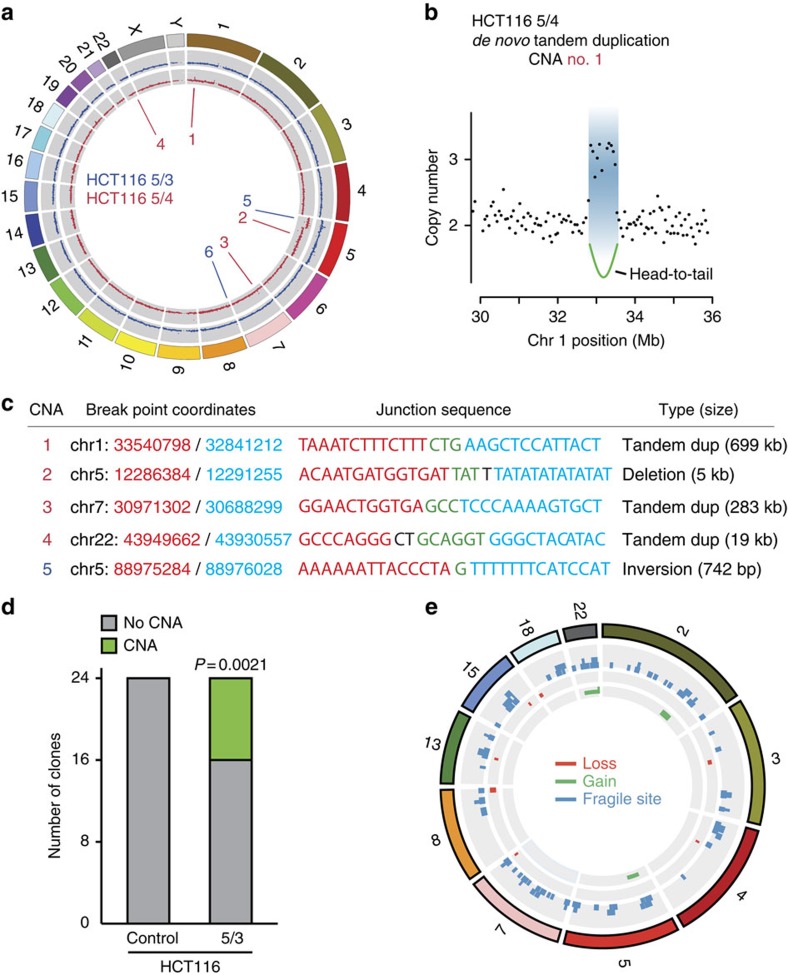
Trisomic and tetrasomic cells acquire *de novo* chromosomal rearrangements. (**a**) Circos plot displaying *de novo* chromosomal rearrangements in HCT116 5/3 (blue) and HCT116 5/4 (red) cells. The outer ring shows all chromosomes. Both grey rings highlight the copy number profile based on mate-pair sequencing data of HCT116 5/3 (blue) and HCT116 5/4 (red) relative to the parental control. *De novo* CNA break point junctions derived from mate-pair sequencing are indicated with red and blue lines in the middle of the Circos plot. CNA No. 6 was found also in the parental cell line. (**b**) Example of a *de novo* 699 kb tandem duplication identified in HCT116 5/4 cells (CNA no. 1). (**c**) Break point junction sequences obtained by Sanger sequencing of *de novo* chromosomal rearrangement break points from **a**. The two chromosomal loci joined together are indicated in red and blue. Microhomology at the junction sequences is highlighted in green. For full results, see Supplementary Fig. 4b. (**d**) Bar plot depicting the number of clonal lines with and without *de novo* CNAs identified by SNP-array profiling. Twenty-four clonal lines were derived from HCT116 5/3 and parental control, respectively. For full results, see Supplementary Figure 5. (**e**) Circos plot depicting the CNAs identified in the clonal trisomic lines. Only affected chromosomes are visualized. Copy number losses are marked in red, gains in green, and known fragile sites in blue.

We hypothesized that the replication stress caused by the presence of extra chromosomes might induce stably inherited genomic rearrangements in human cells. To determine whether this is the case, we performed mate-pair next-generation sequencing (NGS) of the parental cell line HCT116 and cognate trisomic and tetrasomic cell (HCT116 5/3 and HCT116 5/4). Comparison with the control cell line revealed two unique chromosomal rearrangements in the trisomic line and four in the tetrasomic line on five different chromosomes (Fig. 4a). Verification by PCR using primers designed to amplify the break point junctions confirmed that five of the six rearrangements have occurred *de novo* in the aneuploid cells (Supplementary Fig. 4a). The rearrangements involved three head-to-tail tandem duplications, one tail-to-head deletion and one tail-to-tail inversion (Fig. 4b,c). Sanger sequencing of the break point junctions revealed microhomologies in all cases, indicative of replication-mediated rearrangement formation (Fig. 4c and Supplementary Fig. 4b)^31^.

To determine the rate of *de novo* chromosomal rearrangements in aneuploids and in parental cells, we generated two sets of 12 clonal cell lines, each originating either from a single HCT116 5/3 cell or from a single HCT116 cell. After 30 cell doublings, DNA was isolated from each clonal cell line and subjected to SNP-array analysis (Supplementary Fig. 4c). Eight out of the 24 trisomic clonal lines contained *de novo* copy number aberrations (CNAs), whereas none were detected in the 24 clonal cell lines derived from the parental HCT116 cells (Fig. 4d). In total, we identified 12 CNAs on 7 different chromosomes that include a gain of chromosome 7, four duplications and seven deletions ranging from 105 kb to several megabases (Fig. 4e, Supplementary Fig. 5 and Supplementary Table 2). Permutation testing confirmed that the accumulation of *de novo* CNAs among HCT116 5/3 as compared with HCT116 cells was unlikely to have occurred by chance (*P* value=0.0021). By comparison of the identified break point junctions with previously documented common fragile sites, we found that one site overlapped with the fragile sites mapped in the HCT116 cell line after treatment with the replication inhibitor aphidicolin^32^, and 8 out of the 12 CNAs were mapped to fragile sites identified in lymphocytes^33^ (Fig. 4e and Supplementary Table 2). Taken together, the presence of extra chromosomes significantly increases the occurrence of CNAs in human cells. The break point sequences, the types of identified chromosomal rearrangements and their frequent overlap with common fragile sites indicate that they arose due to defects during DNA replication.

### Reduced MCM2-7 levels contribute to genomic instability

**Figure 5:**
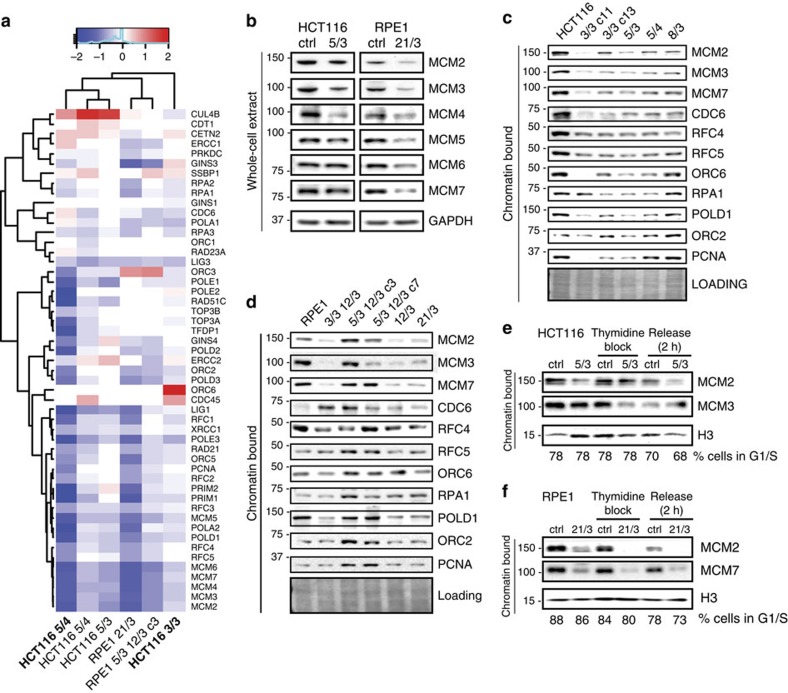
The abundance of replication factors is decreased in response to extra chromosomes. (**a**) Unsupervised hierarchical clustering of the protein abundance fold changes (calculated log2 aneuploid-to-diploid ratio) of factors assigned to KEGG (Kyoto Encyclopedia of Genes and Genomes)-defined term replication; manually curated. HCT116 5/4 and HCT116 3/3 marked in the figure in bold are cell lines previously constructed by Minoru Koi; these cell lines were used only for the global proteome analysis. See Methods for more details. (**b**) Immunoblotting of subunits of the replicative helicase MCM2-7 in whole-cell extracts from trisomic cell lines and their respective controls. (**c**,**d**) Levels of chromatin-bound replication proteins in the parental cell lines and the trisomic and tetrasomic cell lines. Note that MCM2, 3 and 7 are downregulated in all cell lines with extra chromosomes except for RPE1 5/3 12/3 c3. Ponceau staining was used as loading control. (**e**,**f**) Levels of chromatin-bound subunits of the MCM2-7 helicase in asynchronous and synchronized cells in HCT116 5/3 and RPE1 21/3, and respective controls.

The presence of extra chromosomes triggers global and highly conserved expression changes that affect a large number of cellular pathways^20^,^21^. We asked whether these expression changes might explain the phenotypes observed in aneuploid cells. To this end, we analysed protein expression data previously generated using quantitative mass spectrometry^4^. Unsupervised hierarchical clustering revealed downregulation of several replication factors in aneuploid cells (Fig. 5a). Strikingly, expression of all six subunits of the replicative helicase MCM2-7 (hereafter MCM) was consistently and significantly decreased in all analysed cells. MCM is a heterohexameric DNA helicase required for licensing of replication origins and for replication progression. Insufficient licensing owing to low levels of MCM helicase impairs the activation of dormant origins under replication stress conditions^34^,^35^. Immunoblotting confirmed a general 20–50% decrease in all subunits of MCM helicase in HCT116 5/3 and RPE1 21/3 compared with control cells (Fig. 5b). Strikingly, a decrease of MCM2, MCM3 and MCM7 abundance was observed in 9 out of 10 HCT116- and RPE1-derived aneuploidies, regardless of the specific karyotype (Fig. 5c,d). Only chromatin-bound MCM contributes to the activation of replication origins; immunoblotting of MCM2, MCM3 and MCM7 from the chromatin fraction in both asynchronous and synchronized cells confirmed that trisomic and tetrasomic cells load significantly less helicase on DNA than cognate controls (Fig. 5e,f and Supplementary Fig. 6). It should be noted that immunoblotting confirmed downregulation of additional replication proteins, but none of them as consistently as the subunits of the helicase MCM (Fig. 5c,d).

**Figure 6:**
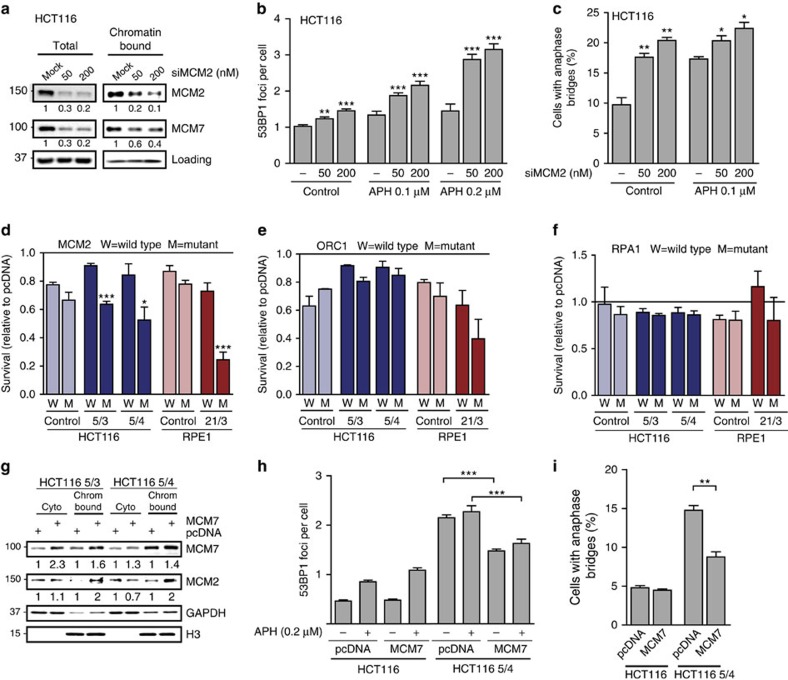
The accumulation of DNA damage is sensitive to abundance changes of MCM2-7 subunits. (**a**) Levels of total and chromatin-bound MCM2 and MCM7 in parental HCT116 upon siRNA-mediated depletion of MCM2. (**b**) Accumulation of 53BP1 and (**c**) anaphase bridges in HCT116 upon depletion of MCM2 with and without replication stress. All plots show mean±s.e.m. of three independent experiments, at least 500 cyclin A-negative cells or 50 anaphases were scored in each experiment. (**d**) Survival rates of HCT116, RPE1 and their trisomic and tetrasomic derivatives upon overexpression of wild-type and mutant alleles of MCM2 (MCM-457A). (**e**) Survival rates of HCT116, RPE1 and the trisomic and tetrasomic derivatives upon overexpression of wild-type and mutant alleles of ORC1 (ORC1ΔBAH). (**f**) Survival rates of HCT116, RPE1 and the trisomic and tetrasomic derivatives upon overexpression of wild-type and mutant alleles of RPA1 (RPA1 L221P). Survival rates were normalized to the control (pcDNA transfected sample). All plots show mean+s.e.m. of three independent experiments; nonparametric *t*-test; *P<0.05, **P<0.01, ***P<0.001. (**g**) Levels of total and chromatin-bound MCM2 and MCM7 in HCT116 5/3 upon transient overexpression of MCM7. (**h**) Formation of 53BP1 foci and (**i**) accumulation of anaphase bridges in HCT116 and HCT116 5/4 upon transient overexpression of MCM7. One representative plot of three independent experiments is shown. Nonparametric *t*-test; *P<0.05, **P<0.01, ***P<0.001.

We hypothesized that the low levels of MCM2-7 contribute to the replication defects observed in aneuploid cells. To test this hypothesis, we partially depleted MCM2 by short interfering RNA (siRNA) in parental HCT116 and RPE1. The abundance of another subunit, MCM7, conjointly decreased, indicating a decrease of the entire complex (Fig. 6a). The partial depletion of MCM2 was sufficient to trigger accumulation of 53BP1 foci and an increased frequency of anaphase bridges in the presence or absence of aphidicolin in HCT116 (Fig. 6b,c) as well as in RPE1 (Supplementary Fig. 7a–c). Other replication factors such as CDC6, ORC2 and RPA1 were also partially downregulated in some cell lines with extra chromosomes; however, downregulation of CDC6 and ORC2 in control cells did not trigger accumulation of 53BP1 foci and anaphase bridges, whereas partial downregulation of RPA1 caused high levels of DNA damage (Supplementary Fig. 7d–i). To further test which of the downregulated replicative factors are limiting in cells with extra chromosomes, we transfected both diploid cells and respective trisomic and tetrasomic cell lines with plasmids carrying either functional or mutant alleles of MCM2, ORC1 and RPA1. The mutant protein MCM2-457A cannot be phosphorylated by Dbf4-dependent kinase (DDK) kinase, thereby rendering replication firing inefficient^36^. ORC1ΔBAH shows decreased binding to DNA^37^, and RPA1 L221P impairs DNA replication and leads to accumulation of DNA damage^38^. None of the mutant alleles exert detrimental effects in the presence of the endogenous wild-type allele; however, they are toxic for a cell in the absence of the wild-type protein. We found that overexpression of the MCM2-457 A allele was highly toxic in trisomic and tetrasomic cell lines, but showed no effect in the control cell lines (Fig. 6d). In contrast, overexpression of ORC1ΔBAH and RPA1 L221P mutants did not impair the proliferation of any of the aneuploid cell lines significantly more than overexpression of the wild-type allele (Fig. 6e,f). We conclude that the MCM helicase is the limiting factor for replication in cells with extra chromosomes.

Finally, we asked whether increasing the levels of MCM2-7 can rescue the accumulation of DNA damage in aneuploid cells. To this end, we transiently transfected HCT116 5/3 and 5/4 with either empty pcDNA vector or with a version carrying pCMV-MCM7. The levels of the chromatin-bound MCM7 as well as MCM2 increased in cells transfected with pCMV-MCM7 in comparison with control cells (Fig. 6g). Importantly, the increase in the MCM2-7 abundance resulted in a significant decrease in 53BP1 foci formation as well as lower occurrence of anaphase bridges in four different cell lines with extra chromosomes (HCT116 3/3, 5/3, 5/4 and RPE1 21/3), but did not affect the control HCT116 or RPE1 (Fig. 6h,i and Supplementary Fig. 8a,b). Overexpression of other replication factors (PolD1,4, RPA1,2,3 and CDC6) or control renilla luciferase did not affect the accumulation of 53BP1 foci (Supplementary Fig. 8c,d). Supplementing the medium with a high concentration of DNA synthesis precursors showed a mild rescue of the 53BP1 foci formation in only one cell line and no rescue of the EdU incorporation (Supplementary Fig. 8e–g). Altogether, our findings strongly suggest that the presence of extra chromosomes causes replication stress and genomic instability in human cells owing to downregulation of components of the MCM2-7 helicase.

## Discussion

Here we demonstrate for the first time that the addition of even a single chromosome to human cells promotes genomic instability by increasing DNA damage and sensitivity to replication stress. Replication stress is emerging as one of the primary causes of genomic instability during early stages of tumorigenesis^9^,^10^,^11^. Our findings provide a rationale for how random missegregation of a single chromosome might contribute to genomic instability and potentially to early events in tumorigenesis.

To systematically analyse the effect of trisomy and tetrasomy on genomic instability in human cells, we analysed five different trisomies and tetrasomies derived from HCT116 and five RPE1-derived cell lines. Remarkably, we found strikingly similar phenotypes regardless of the identity of extra chromosomes. Indeed, the presence of extra chromosomes leads to (1) accumulation of DNA damage likely originating from replication errors (Figs 1 and 2); (2) increased sensitivity to additional replication stress (Fig. 3a,b); and (3) abnormal DNA replication (Supplementary Fig. 3 and Fig. 3c). The uniformity of the phenotypes and the fact that the degree of some defects tends to scale with the amount of the added DNA suggest that the presence of an additional chromosome alone is the culprit, which causes the genetic instability in these cells.

The altered DNA replication and increased DNA damage affects genomic stability in trisomic and tetrasomic cell lines, as documented by the increased occurrence of *de novo* CNAs. These structural rearrangements were caused by the presence of extra chromosomes, as parental HCT116 did not show any CNAs after the same number of generations (Fig. 4a,d). The CNAs affected all chromosomes evenly, and no enrichment on the extra chromosome was observed. The pattern of identified CNAs, showing a bias towards deletions and tandem duplications, and the occurrence of microhomology at the break point junctions further support the idea that the addition of extra chromosomes specifically impairs DNA replication, thus inducing replication-induced breaks at stalled/arrested replication forks^31^.

What causes the genetic instability in response to extra chromosomes? It has been shown that trisomy and tetrasomy cause genome-wide gene expression changes; one of the most prominent features is a strong downregulation of factors related to DNA replication^20^,^21^. In particular, the subunits of the replicative helicase MCM2-7 are significantly downregulated in all aneuploid cell lines that we analysed (Fig. 5). Loading of the MCM helicase together with ORC2-5 proteins, CDT1 and CDC6, is essential for licensing of origins of replication, and MCM is also required for fork progression. Replication dynamics are unaffected by partial depletion of MCM under normal growth conditions as the number of MCM complexes loaded onto the DNA is greater than the number of the actual active origins^34^,^35^. Under replication stress, however, a reduction in the level of MCM proteins leads to a decreased licensing of dormant origins that are needed to allow completion of the DNA replication. Accordingly, cells are markedly sensitive to MCM dosage changes. Several lines of evidence suggest that the observed decrease of 20–50% of MCM2-7 abundance indeed contributes to the genomic instability observed in cells with extra chromosomes. First, depletion of MCM in control cells to levels equivalent to those observed in tri- and tetrasomes results in comparable increase in DNA damage foci, whereas partial depletion of ORC2, RPA1 and CDC6 did not cause similar phenotypes (Fig. 6a–c and Supplementary Fig. 7a–i). Second, depletion of MCM3 was previously shown to lead to an increase in fragile site instability^39^, similarly to cells with extra chromosomes that show increased levels of fragile sites on metaphase spreads under replication stress conditions. Moreover, fragile site instability may originate from origin paucity and/or from a failure in dormant origin activation^39^,^40^. Third, mouse models homozygous for an MCM4^*chaos*^ mutation that impairs the stability of the MCM4 subunit, or an MCM2^IRES-CreERT2^ mutation that reduces the levels of MCM2, suffer from impaired proliferation, slow replication rates and genomic instability^41^,^42^,^43^, comparable to trisomic and tetrasomic cell lines in this study. Fourth, overexpression of mutant MCM2 protein is toxic in cells with extra chromosomes but not in control cell lines, whereas mutant RPA1 and ORC1 do not affect the proliferation of aneuploid cell lines more than overexpression of the wild-type allele, suggesting that indeed the MCM complex is the limiting DNA replication factor in trisomic and tetrasomic cells. Finally, we show that elevating the levels of MCM2-7 by exogenous overexpression alleviates the replication-induced defects and DNA damage in aneuploid cells (Fig. 6g,h). The effect of MCM overexpression was stronger in trisomies and tetrasomies with a relatively high levels of genomic instability, such as HCT116 5/4 or RPE1 21/3, than in HCT116 5/3 with a rather mild defect. This is in agreement with the idea that high levels of MCM2-7 helicase become critical in cells with elevated replication stress, and also suggest that other factors play a role in genomic instability of aneuploids as well.

Alternative explanations for the observed phenotypes are possible. For example, overexpression of a specific gene located on a supernumerary chromosome might impair maintenance of genome stability. However, the fact that the trisomies and tetrasomies of five different chromosomes (3, 5, 8, 12 and 21) lead to strikingly similar phenotypes does not support this explanation. Another possibility is that the extra DNA simply titrates away a limiting factor, thus slowing down replication. This hypothesis implies that additional DNA and its replication alone should trigger the same phenotypes. However, transcriptional silencing of chromosome 21 using XIST expression in cells with trisomy 21 results in improved proliferation^44^, similarly as the full removal of chromosome 21 by counter-selection against TKNeo^45^. Moreover, haploid yeast with extra chromosomes show genetic instability, but the phenotype is not observed in cells that contain a yeast artificial chromosome that can be replicated, but not transcribed by these cells^24^. Thus, we propose that it is not the need to replicate additional DNA, but rather the pathway deregulation and stoichiometric imbalances caused by aneuploidy that promote the observed genetic instability.

Why the replication factors are downregulated in response to aneuploidy remains enigmatic. This question goes together with the observation that aneuploidy causes global expression changes, with DNA replication being among the most consistently downregulated pathways across several analysed cell lines and species^20^,^23^. One possibility is that some pathways are deregulated due to the proteotoxic stress and protein folding defect in trisomic and tetrasomic human cells^23^. Accordingly, several factors involved in DNA repair and replication are well-characterized clients of molecular chaperones^46^. Alternatively, activity of E2F transcription factors that control expression of MCM subunits or DNA polymerases^47^ might be altered in response to trisomy and tetrasomy. Interestingly, recent results revealed that ageing haematopoietic stem cells in mice suffer from replication stress, cell cycle defects and chromosomal aberrations owing to a decreased expression of MCM helicase^48^. The reasons for the downregulation of MCM in ageing haematopoietic stem cells remain unclear. Together with our observations in trisomic and tetrasomic cells, we suggest that the expression levels of MCM helicase are a highly sensitive measure of cellular stress.

In summary, our results show that addition of even a single chromosome renders human cells sensitive to replication stress and elevates the occurrence of CNAs in human cells, thereby suggesting a novel mechanism for how chromosome segregation errors may fuel genomic instability. The observed phenotypes may provide new insights into the causes of developmental defects associated with congenital trisomies and might help address the question of why patients with Down's syndrome show altered spectra of malignant diseases with an increase in haematological malignancies and a decrease in solid tumours 49. Markedly, cancer cells often express DNA replication factors including the MCM helicase at high levels, and MCM2 and MCM7 are considered as biomarkers and potential therapeutic targets for cervical, colorectal and other tumours (for example, refs 50, 51, 52). Identification of the mechanisms that allow cancer cells to adapt to aneuploidy by elevating the expression of replication factors might illuminate novel opportunities for cancer therapy.

## Methods

### Cell lines

The cell line RPE1 hTERT (redeferred to as RPE1) and RPE1 hTERT H2B-GFP were a kind gift from Stephen Taylor (University of Manchester, UK). HCT116 H2B-GFP was generated by lipofection (FugeneHD, Roche) of HCT116 (American Type Culture Collection no. CCL-247) with pBOS-H2B-GFP (BD Pharmingen) according to manufacturer's protocols. Trisomic and tetrasomic cell lines were generated by microcell-mediated chromosome transfer as described below. The donor mouse cell lines A9(Neo3), A9(Neo5), A9(Neo8) and A9(Neo21) were purchased from the Health Science Research Resources Bank (HSRRB), Osaka 590-0535, Japan. All cell lines were maintained at 37 °C with 5% CO_2_ atmosphere in Dulbecco's Modified Eagle Medium (DMEM) containing 10% fetal bovine serum, 100 U penicillin and 100 U streptomycin. The cell lines HCT116 3/3, HCT116 H2B-GFP 5/3, HCT116 H2B-GFP 5/4, RPE1 5/3 12/3, RPE1 H2B-GFP 21/3 and A9(Neo5) were grown supplemented with 400 μg ml^−1^ G418. The cell line HCT116 5/4 as well as the cell lines stably transfected with H2B-GFP were grown in media supplemented with 6 μg ml^−1^ blasticidin S. Before each experiment, aneuploid cells were grown one passage in medium without the antibiotic selection. All the cell lines were tested for mycoplasma contamination. The cell lines HCT116, HCT116 5/4 and HCT116 3/3 were kindly provided by Minoru Koi, Baylor University Medical Centre, Dallas, TX, USA. These cell lines were used only for the global proteome analysis (Fig. 5a). For nucleoside incorporation analysis, the culture medium was supplemented with adenosine (Sigma A9251), cytidine (Sigma C4654), uridine (Sigma U3750) and guanosine (Sigma G6752) to a final concentration of 50μM for 48 h. In order to synchronize cells in S-phase, HCT116- and RPE1-derived cells were cultured for 30 h in 2 mM thymidine, washed three times in PBS and were released into the standard DMEM medium.

### Microcell-mediated chromosome transfer

To generate aneuploid HCT116 and RPE1 containing an additional chromosome 3, 5, 8 or 21, microcell fusion (Supplementary Fig. 1a) was performed as follows^53^. In brief, murine donor cells containing additional human chromosome with a resistance gene were treated for 48 h with colchicine (final concentration 60 ng ml^−1^). Donor cells were trypsinized and seeded on plastic bullets. After the cells were attached to the surface, bullets were centrifuged at 27,000*g* for 30 min at 30–34 °C in DMEM supplemented with 10 μg ml^−1^ cytochalasin B. Cell pellets were resuspended in serum-free DMEM and filtered to clear suspension from mouse cells. Filtered microcells were mixed with phytohaemagglutinin (PHA-P) and added to the recipient cell line HCT116 H2B-GFP, RPE1 or RPE1 H2B-GFP. Fusion of microcells with the recipient cells was facilitated by polyethylene glycol 1500 treatment. Cells containing the additional human chromosome were selected in medium supplemented with 400 μg ml^−1^ G418 or with hygromycin. The chromosome 12 in trisomic and tetrasomic cell lines derived from RPE1 carries no gene coding for resistance. The cell lines were obtained because of a spontaneously occurring aberration. Parental cell line HCT116 stably expressing histone H2B-GFP was used for cell lines marked HCT116 5/3 (trisomy 5) and HCT116 5/4 (tetrasomy 5); parental cell line RPE1 (human retinal pigment epithelial cell line, hTERT immortalized) was used for RPE1 5/3 12/3 (trisomy 5, 12); parental cell line RPE1 stably expressing histone H2B-GFP was used for RPE1 21/3 (trisomy 21). Cells were grown in DMEM GlutaMax (Gibco) supplemented with 10% fetal bovine serum and 5% penicillin–streptomycin under standard conditions. Clonal populations arising from single cell after the MMCTs were isolated and further expanded. Subsequently, chromosome spreads combined with chromosome painting were performed (see below). Clonal populations that gained the expected extra chromosomes were further expanded for three passages and at least 15 vials were frozen in liquid nitrogene. Simultaneously, a sample was subjected to SNP-array analysis or array comparative genomic hybridization (aCGH). Only cells with fully analysed karyotypes were used for the experiments. All experiments were always performed from the same passage vial to minimize clonal effects, and the cells were kept in culture for maximum three passages, unless otherwise stated. No selection for the extra chromosome was applied for 24 h before each experiment. For further details, see Supplementary Table 1.

### Preparation of chromosome spreads

Cells were grown to 70–80% confluency, treated with 50 ng ml^−1^ colchicine for 3–5 h, collected by trypsinization and centrifuged at 250*g* for 10 min. Pellets were resuspended in 75 mM KCl and incubated for 10–15 min at 37 °C. After centrifugation at 150*g* for 10 min, cell pellets were resuspended in 3:1 methanol/acetic acid to fix the cells. Cell pellets were washed several times in 3:1 methanol/acetic acid, spread on a wet glass slide and air dried at 42 °C for 5 min.

### Chromosome painting

Chromosome spreads were prepared as described above. Each sample was labelled with probes for two different chromosomes: a transferred chromosome and a control chromosome. Probes (Chrombios GmbH, Raubling, Germany) for chromosomes 2, 3, 5 and 21 were tagged with TAMRA, FITC, Cy-5 and TAMRA, respectively. The chromosomes were labelled according to the manufacturer's instructions and counterstained with 4′,6-diamidino-2-phenylindole (DAPI). Images were obtained by a fully automated Zeiss inverted microscope.

### Array comparative genomic hybridization

Genomic DNA for aCGH analysis was extracted using the Qiagen Gentra Puregene Kit according to manufacturerŕs instructions. The aCGH analysis was performed by IMGM Laboratories, Martinsried, Germany. Commercially available human genomic DNA (Promega) was used as a reference sample for all 4 × 44K array-based analyses (HCT116, HCT116 3/3, HCT116 5/4, HCT116 H2B-GFP 5/3, HCT116 H2B-GFP 5/4, RPE1 and RPE1 5/3 12/3). RPE1 H2B-GFP 21/3 was analysed by SurePrint 4 × 180K G3 Human CGH Microarray. Genomic DNA extracted from HCT116 was used as a reference for the high-density CGH analysis of HCT116 5/4 by the 2 × 400K array. gDNA concentration and DNA absorbance ratio (260 nm/280 nm) were measured by NanoDrop ND-1000 ultraviolet–visible spectrophotometer (PeqLab). An amount of 1 μg of gDNA was used for each reaction. gDNA integrity was tested on an 1.0% agarose gel stained with ethidium bromide. A measure of 1.0 μg gDNA of each sample was subjected to restriction digestion with a combination of Alu I and Rsa I restriction enzymes. The digested gDNA samples were directly labelled with exo-Klenow fragments and random primers by incorporation of Cy-5 dUTP (dUTP=2′-deoxyuridine 5′-triphosphate) for the experimental samples and Cy-3 dUTP for the reference samples (Genomic DNA Enzymatic Labeling Kit, Agilent Technologies). After purification, each experimental sample was combined with its respective reference sample and hybridized to respective arrays. All microarrays have been washed with increasing stringency using Oligo aCGH Wash Buffers (Agilent Technologies) followed by drying with acetonitrile (Sigma-Aldrich). Fluorescent signal intensities for both dyes were detected with Scan Control 8.4.1 Software (Agilent Technologies) on the Agilent DNA Microarray Scanner and extracted from the images using Feature Extraction 10.5.1.1 Software (Agilent Technologies). The software Feature Extraction 10.5.1.1 as well as the software Genomic Workbench 5.0.14 was used for quality control, statistical data analysis and visualization. Raw microarray data were normalized. ADM-2 aberration algorithm was applied together with centralization algorithm. Aberrations for all samples were filtered from the whole-genome data and analysed based on a threshold of log2 ≥0.39 for amplifications and log2 ≤−0.30 for deletions with at least five consecutive aberrant probes.

### Mate-pair NGS

Whole-genome mate-pair sequencing of HCT116 5/3 and HCT116 5/4, and the respective parental control was performed as described^54^. In brief, DNA was sheared to 3 kb fragments, followed by mate-pair library preparation according to the SOLiD 5500 Long Mate-Pair procedure (Life Technologies). Each library was sequenced in 2 × 50 bp configuration on a single lane of a SOLiD 5500 instrument. Colour space reads were mapped to the human genome (GRCh37) using BWA version 0.5.0. Discordant mate pairs were detected and clustered for all samples together as described^54^. Unique clusters of discordant mate-pair reads were identified by filtering out clusters supported by read pairs in >1 sample. Based on discordant mate-pair clusters, we designed primers for break point junction amplifications using Primer3 software. PCR amplification was performed using Amplitaq Gold polymerase (Life Technologies) under standard conditions. Sanger sequencing was performed to identify break point junction sequences at nucleotide resolution.

### SNP-array profiling

Human CytoSNP-12 bead chip arrays (Illumina) for detecting CNAs were used in clonal aneuploid and control cell lines. Array hybridization was performed according to the manufacturer's recommendations. CNAs were called using Nexus software (version 7.5.1) with standard settings. To identify unique CNAs in clonal cell lines, we used the Nexus call coordinates and removed all calls of the same type with a reciprocal overlap of at least 60%. Unique CNA calls were manually curated based on copy number and allele frequency profiles. Permutation testing was performed to calculate the significance of the difference in the distribution of unique CNAs among HCT116 (controls) and HCT116 5/3 (cases) clonal lines. We generated 1,000,000 permutations of the case/control labels per set and determined the *P* value as the proportion of randomizations where all 12 unique CNAs were found in HCT116 5/3 clonal lines.

### Mapping the fragile sites

Genomic positions of common and rare fragile sites were obtained from refs 32, 33. The coordinates matching the chromosomal bands with fragile sites were overlapped with CNA break points using BEDTools^55^.

### Immunofluorescence labelling

Cells were seeded in glass-bottomed black well 96-well plates (Greiner Bio-One, Frickenhausen, Germany), and when necessary treated with aphidicolin (Sigma, 2 μM for 24 h). At the time of evaluation (2 days after plating), the cells were typically 80% confluent. For 53BP1 foci quantification, cells were fixed with 3.7% buffered formaldehyde (12 min at room temperature), permeabilized in 0.2% Triton X-100 in PBS (5 min) and co-immunostained with 53BP1 (1:1,000; Santa Cruz sc-22760) and cyclin A2 (1:200; Abcam ab16726) overnight at 4 °C, followed by a secondary anti-rabbit antibody (Alexa Fluor 647 1:1,000; Jackson ImmunoResearch 711-605-152) and anti-mouse antibody (Alexa Fluor 594 1:1,000; Jackson ImmunoResearch 715-858-150) for 1 h at room temperature. To detect UFBs, the cells were fixed with 4% paraformaldehyde (15 min at −20 °C), permeabilized in 0.5% Triton X-100 in PBS (20 min on ice), blocked (10% FCS and 0.1% Triton X-100) for 45 min at room temperature and immunostained with antibodies against BLM (1:200; Santa Cruz sc-7790) overnight at 4 °C, followed by the secondary anti-goat antibody (Alexa Fluor 647 1:1000; Jackson ImmunoResearch 711-605-152) for 1 h at room temperature. For mitotic error analysis, mitotic cells were collected by mitotic shake-off, fixed in glass-bottomed black well 96-well plates with 100% ice-cold methanol for 10 min and blocked (10% fetal calf serum and 0.1% Triton X-100) for 45 min at room temperature. Cells were co-immunostained at 4 °C overnight with antibodies for α-tubulin (1:500; Sigma T6199) and CREST (1:1,000; Immunovision HCT-0100) followed by a secondary anti-human antibody (Alexa Fluor 647 1:1,000 and Molecular Probes A-21445) and anti-mouse antibody (Alexa Fluor 594 1:1,000 and Jackson ImmunoResearch 715-858-150) for 1 h at room temperature. The DNA was counterstained when necessary by SYTOX Green (1:5,000, Invitrogen S7020).

### Microscopy and image analysis

Confocal microscopy was performed using a fully automated Zeiss inverted microscope (AxioObserver Z1) equipped with a MS-2000 stage (Applied Scientific Instrumentation, Eugene, OR), the CSU-X1 spinning disk confocal head (Yokogawa) and LaserStack Launch with selectable laser lines (Intelligent Imaging Innovations, Denver, CO). Image acquisition was randomized and at least 12 non-overlapping fields were captured for each well using a CoolSnap HQ camera (Roper Scientific) and a × 40 air objective (Plan Neofluar × 40/0.75) under the control of the Slidebook software (version 5.0; Intelligent Imaging Innovations). The numbers of 53BP1 foci per nucleus were determined by an automated pipeline using the public domain, free software CellProfiler (http://www.cellprofiler.org/). Image processing was performed for the whole data set using standard CellProfiler modules for illumination correction, segmentation, masking and thresholding. The nuclei were detected using the 473 channel. Out-of-focus images were manually excluded from the data set. Within the detected cyclin A2-negative nuclei, 53BP1 foci number was measured using the Alexa 647 fluorescent signal. The average number of 53BP1 foci per nucleus was determined for each cell line. All experiments were performed in at least three biological replicates, and a minimum of 500 cells were analysed in each replicate. The statistical analysis of the data was performed using Prism (GraphPad Software, Inc.) and significance was calculated by nonparametric two-tailed *t*-test or *χ*^2^-test.

### Metaphase preparation for chromosomal fragility analysis

Cells were harvested after a 40-min treatment with 100 ng ml^−1^ colchicine followed by a 30-min incubation in 0.4% KCl at 37 °C and multiple changes of 3:1 methanol:acetic acid fixative. Cells were then dropped onto slides and stained with propidium iodide. Gaps and constrictions on metaphase chromosomes were analysed using a Nikon fluorescent microscope. At least 50 metaphases for each condition were analysed. The significance was analysed by Fisher's exact test with defined classes of cells with ≤5 errors and cells with >5 errors for HCT116; two errors were used as a cut off for RPE1.

### Cell cycle analysis

Following treatment with aphidicolin, the cells were harvested and washed with cold PBS. Cells were fixed in −20 °C 100% methanol and incubated in −20 °C overnight. For FACS analysis, fixed cells were resuspended in PBS containing 0.2 μg μl^−1^ RNase for 30 min, followed by staining with 50 μg ml^−1^ propidium iodide. DNA content was analysed by flow cytometry (Becton Dickinson FACS calibur).

### Global rate of DNA synthesis by EdU incorporation

Two days after plating, cells were treated for 4 h with aphidicolin or hydroxyurea at a final concentration of 0.1 μM and 0.2 mM, respectively. During the last 2 h of treatment, EdU (10 μM) was added to cells. For EdU detection, cells were fixed for 15 min with 3.7% paraformaldehyde, permeabilized for 15 min with 0.1% Triton X-100 in PBS and incubated with EdU Click-iT cocktail (Invitrogen) following the manufacturer's instructions. The DNA was counter-stained with DAPI. All samples were imaged by automatized fluorescence microscopy, and EdU-positive cells were quantified by CellProfiler. The analysis was performed in three biological replicates and at least 1,000 cells were imaged in each replicate.

### Subcellular fractionation and western blotting

Cytoplasmic and chromatin-bound fractions were extracted using a subcellular protein fractionation kit (Thermo Scientific, Rockford, IL) following the manufacturer's instructions. Whole-cell lysates were obtained using RIPA buffer supplemented with protease inhibitors (Roche). An amount of 20 μg of protein was then resolved on 10% polyacrylamide gels and transferred to nitrocellulose membranes using the semi-dry technique. Ponceau staining was performed by incubating the membrane for 5 min in Ponceau S solution (0.2 (w/v) in 1% (v/v) acetic acid). After blocking in low fat, 5% BSA in Tris-Buffered Saline with Tween 20 for 1 h at room temperature, membranes were incubated overnight at 4 °C with the following primary antibodies: MCM2 (1:2,000; Abcam ab4461), MCM3 (1:1,000; Cell Signaling no. 4012), MCM4 (1:1,000; Biorbyt orb32710), MCM5 (1:1,000; Biorbyt orb128349), MCM6(1:1,000; Biorbyt orb48451), MCM7 (1:1,000 Santa Cruz sc-9966), FANCD2 (1:1,000; Santa Cruz sc-20022), POLD1 (1:1,000; Bethyl A304-007A), POLD3 (1:1,000; Bethyl A301-244A), RPA1 (1:1,000; Abcam ab79398), CDC6 (1:1,000; Santa Cruz sc-9964), ORC6 (1:1,000, Santa Cruz sc-32735), ORC2 (1:1,000, Santa Cruz sc-32734), RFC4 (1:1,000; Santa Cruz sc-20996), RFC5 (1:1,000; Bethyl A300-146A), PCNA (1:1,000; Chromotek 16d10) and RPA2 (1:1,000; Abcam ab2175). pRPA2 S33 (1:1,000; Bethyl A300-246A), GAPDH (1:2,000; Cell Signaling no. 2118) and H3 (1:1,000; Millipore 05-499). After incubation with horseradish peroxidase-conjugated secondary antibodies, horseradish peroxidase substrate was added and luminescent signals were quantified using a LAS 3000 instrument (FujiFilm). Protein bands were quantified using ImageJ software. All experiments were performed in at least two biological replicates. Uncropped blots are shown in Supplementary Figs 9 and 10.

### Electroporation

Cells were electroporated using the Amaxa Nucleofector II apparatus according to the manufacturer's instructions for HCT116 and RPE1 cells, respectively. In brief, 1 million cells were resuspended in Cell Line Nucleofector Solution V containing 2 μg of respective plasmids and transferred to cuvettes. For siRNA, cells were resuspended in Cell Line Nucleofector Solution V containing the indicated concentration of siRNA. The previously published sequences were acquired from Eurofins Genomics and are as follows: MCM2 (5′-GGAGCUCAUUGGAGAUGGCAUGGAA-3′); GL2 (5′-CGUACGCGGAAUACUUCGATT-3′), ORC2 (5′-GAUCAGCUAGACUGGAUAGUA-3′), CDC6 (5′-ACUAGAACCAACAAAUGUC-3′) and RPA1 (5′-AACUGGUUGACGAAAGUGGUG-3′). HCT116 cells were electroporated using the D-032 program, and for RPE1 cells the program wags U-017. Co-transfection with a GFP overexpressing vector revealed that ∼50–70% of the cells are transfected in each experiment. All cells were scored for 53BP1 foci and anaphase bridges quantification after transfection.

### Statistical analysis

Statistical analyses were performed using a two-tailed *t*-test, two-tailed Fisher's exact test or *χ*^2^-test as indicated in the corresponding figure legend (*t*-test; *P<0.05, **P<0.01, and ***P<0.001). Values are shown as the mean±s.e.m. of multiple independent experiments. The quantifications were software based (CellProfiler); in other cases, the investigators were blinded to allocation during outcome assessment.

## Additional information

**Accession codes**: The break point junction sequencing data was uploaded to NCBI GenBank under the accession codes KU587988, KU587989, KU587990, KU587991, KU587992. The mate-pair sequencing data is available from the European Nucleotide Archive (ENA; http://www.ebi.ac.uk/ena/) under the accession number PRJEB7596. SNP array data were uploaded to NCBI Gene Expression Omnibus upon the accession number GSE71978.

**How to cite this article:** Passerini, V. *et al.* The presence of extra chromosomes leads to genomic instability. *Nat. Commun.* 7:10754 doi: 10.1038/ncomms10754 (2016).

## Supplementary Material

Supplementary Information Supplementary Figures 1-10 and Supplementary Tables 1-2
