# Supplementary material for: The presence of extra chromosomes leads to genomic instability
Source: Nat Commun. 2016 Feb 15;7:10754. doi: 10.1038/ncomms10754 (PMC4756715; doi:10.1038/ncomms10754)
Supplement: Supplementary Information — Supplementary Figures 1-10 and Supplementary Tables 1-2 [file ncomms10754-s1.pdf]

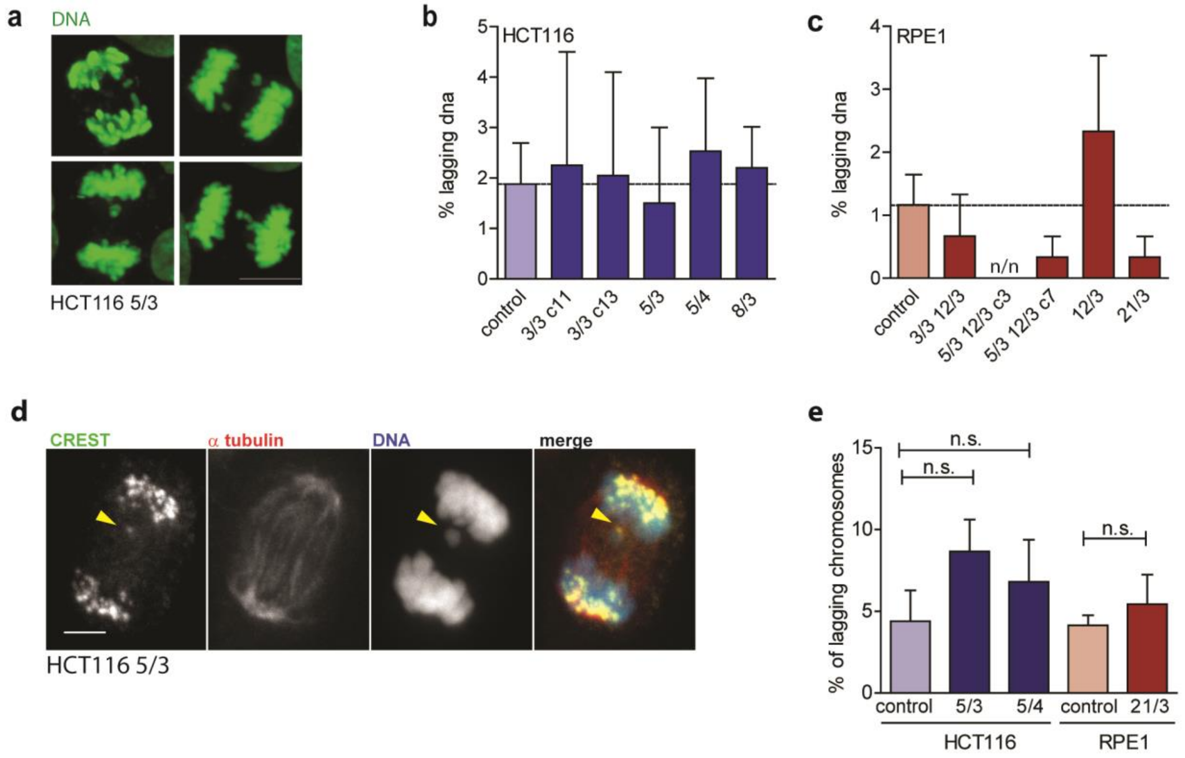

Supplementary Figure 1 Chromosome missegregation in trisomic and tetrasomic cells

(**a**) Example of anaphase cell with a lagging chromosome. Bar = 10 µm (**b**) (**c**) Quantification of lagging chromosomes in diploid controls and the respective trisomic and tetrasomic derivatives. Plots show mean ± SEM of three independent experiments. At least 100 anaphases were scored in each experiment. (**d**) Example of anaphase cell with a lagging chromosome. Bar = 5 µm. (**e**) Percentage of cells with lagging chromosomes scored as DNA mass positive for CREST staining. Plots show mean + SEM of three independent experiments. Non-parametric T-test.

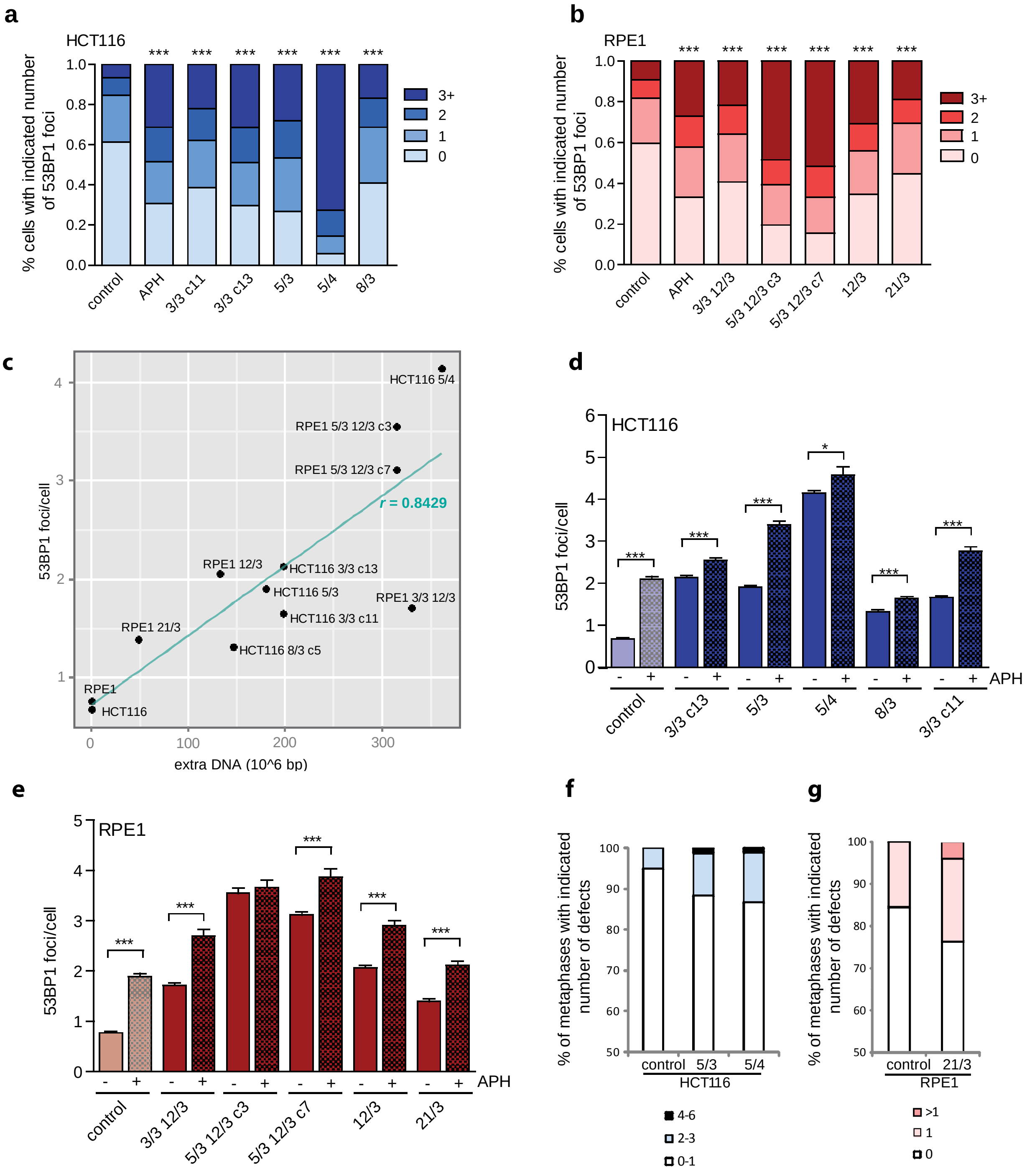

Supplementary Figure 2 Trisomy and tetrasomy elevates DNA damage

(**a**) (**b**) Quantification of % of cells with specific numbers of 53BP1 foci in HCT116 and the trisomic and tetrasomic derivatives (**a**) and RPE1 and the trisomic and tetrasomic derivatives (**b**). Control - parental cell line. APH -parental cell line treated with aphidicolin. Contingency tables were created from 3 independent experiments (n>500) and chi-square test was calculated comparing the number of cells with less than 3 foci or 3 and more foci in control and each trisomic and tetrasomic derivative. (**c**) The number of 53BP1 foci per cell scales with the amount of additional DNA. Note the similarity of the

independent clonal cells lines with the same extra chromosome - HCT116 3/3 c11 and c13 and RPE1 5/3 12/3 c3 and c7. (**d**)(**e**) 53BP1 foci in parental and derived cell lines upon treatment with aphidicolin. Plots show mean ± SEM of three independent experiments, at least 1000 cyclin A-negative cells were scored in each experiment. Non-parametric T test; ***: P<0.001. (**f**) (**g**) Occurence of metaphases with chromosome breaks and other abnormalities in untreated cells. N = 80,78,90,110,76 metaphases for HCT116, 5/3, 5/4, RPE1 and 21/3, respectively, obtained in two independent experiments.

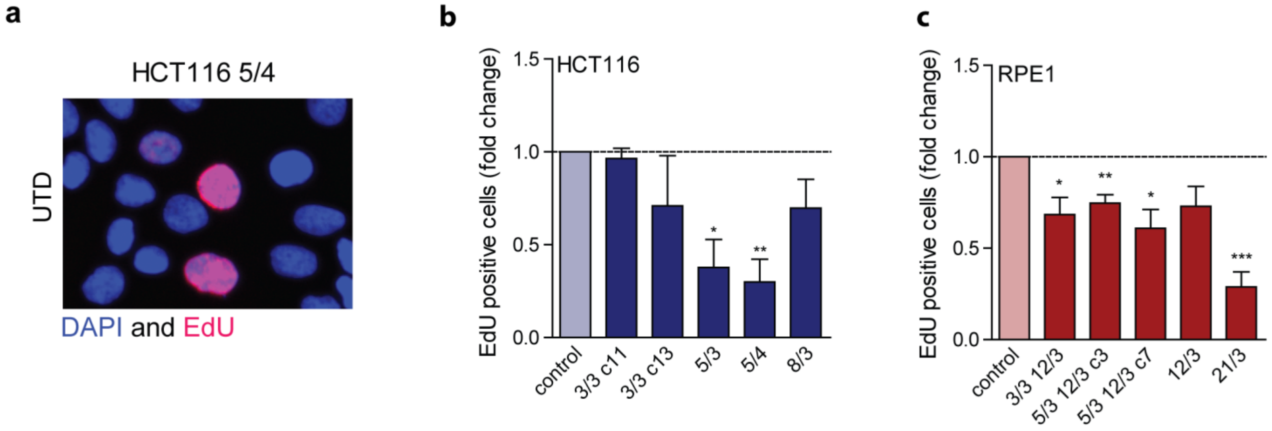

Supplementary Figure 3 Sensitivity of aneuploid cells to replication stress

(**a**) Representative images of HCT116 5/4 cells stained with DAPI and EdU. Cells were grown in the presence of EdU for two hours. (**b**)(**c**) Quantification of EdU-positive cells in control HCT116 and aneuploid derivatives (**b**) and control RPE1 cells and aneuploid derivatives (**c**). All plots show mean ± SEM of three independent experiments, at least 1000 cells were scored in each experiment. Non-parametric T test; * $p<=0.05$, ** $p<=0.01$, *** $p<=0.001$

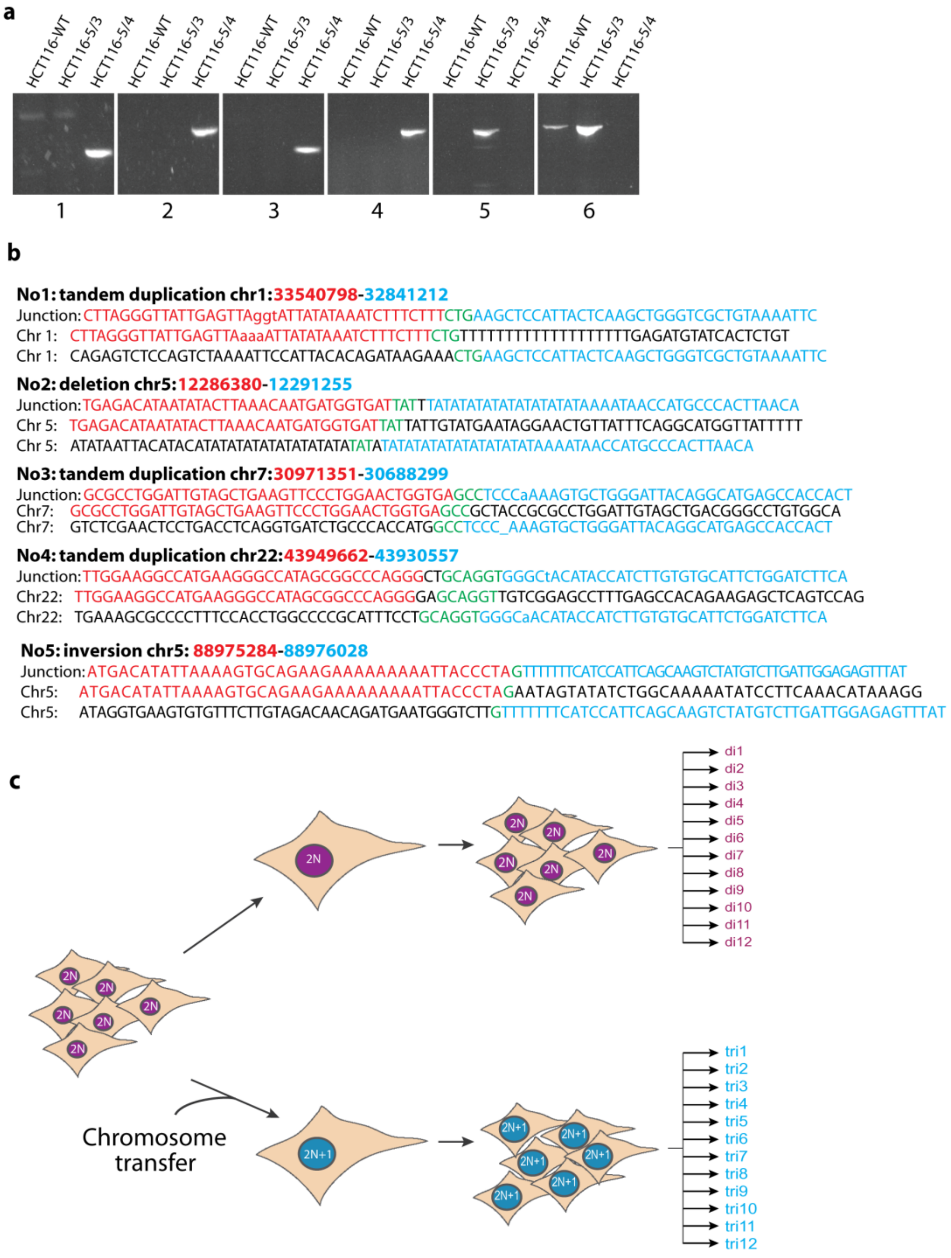

Supplementary figure 4 Mate-pair sequencing and single nucleotide polymorphism arrays to identify chromosomal rearrangements in aneuploid cell lines

(**a**) Analysis of breakpoint junction by PCR for each of the six predicted de novo chromosomal rearrangements. Note that rearrangement 6 was also identified in the parental HCT116 indicating that this rearrangement did not occur de novo. (**b**) Breakpoint junction sequences of five de novo CNAs identified in aneuploid cells.In each case, the upper sequence denotes the breakpoint junction sequence and the two lower sequences denote the genomic regions where both flanks (red and blue) map to. Microhomologies are indicated in green. (**c**) Schematic depiction of the generation of clones derived from single cells that were used for the single nucleotide polymorphism (SNO) profiling. di1 - 12 clones derived from parental cell line HCT116; tri1 -12 clones derived from trisomic cell line HCT116 5/3. Two sets of these experiments were performed, 2x12 clonal cell populations were analyzed for each cell line.

a

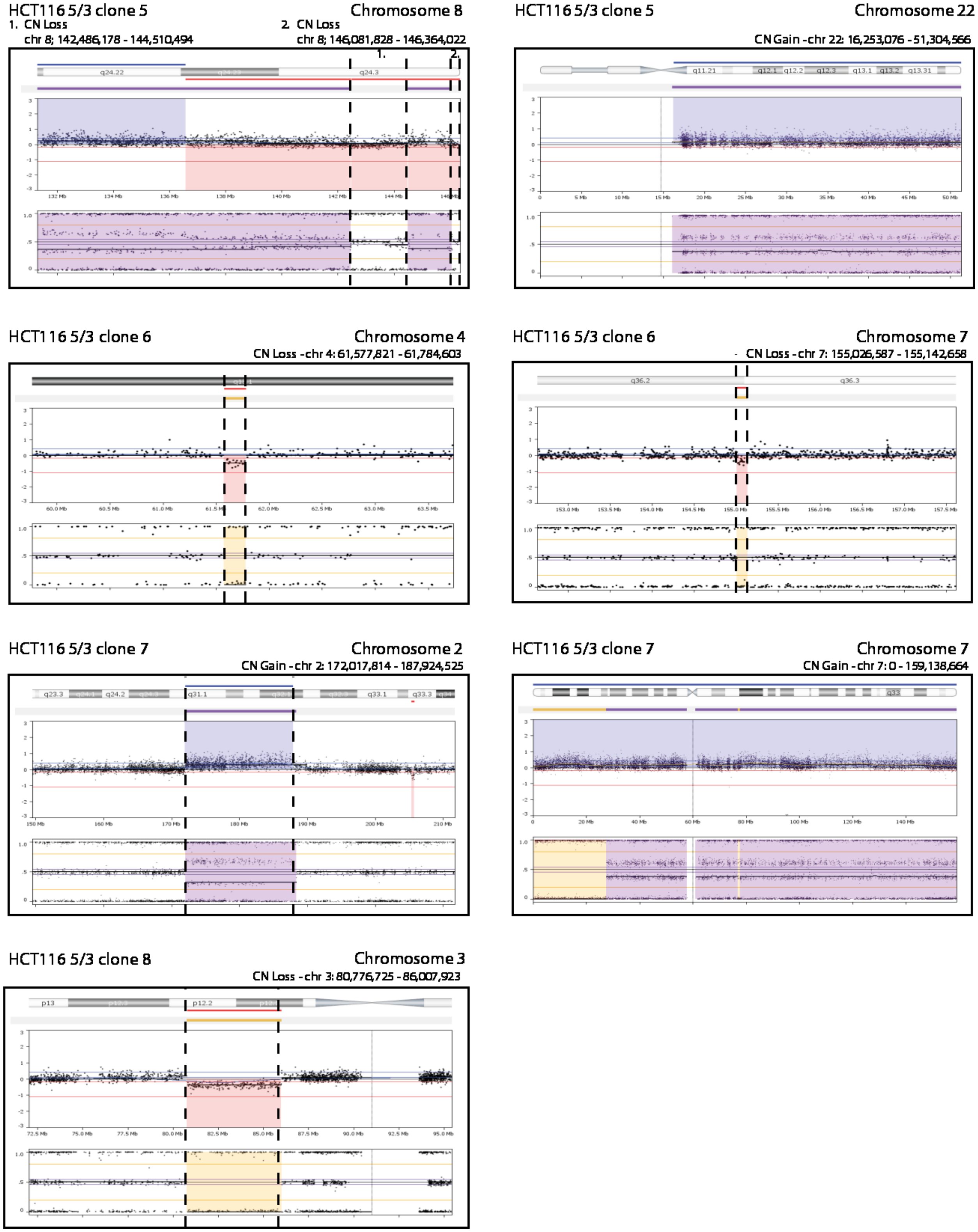

HCT116 5/3 clone 5
Chromosome 8
1. CN Loss
chr 8; 142,486,178 - 144,510,494
2. CN Loss
chr 8; 146,081,828 - 146,364,022
HCT116 5/3 clone 5
Chromosome 22
CN Gain - chr 22: 16,253,076 - 51,304,566
HCT116 5/3 clone 6
Chromosome 4
CN Loss - chr 4: 61,577,821 - 61,784,603
HCT116 5/3 clone 6
Chromosome 7
CN Loss - chr 7: 155,026,587 - 155,142,658
HCT116 5/3 clone 7
Chromosome 2
CN Gain - chr 2: 172,017,814 - 187,924,525
HCT116 5/3 clone 7
Chromosome 7
CN Gain - chr 7: 0 - 159,138,664
HCT116 5/3 clone 8
Chromosome 3
CN Loss - chr 3: 80,776,725 - 86,007,923

b

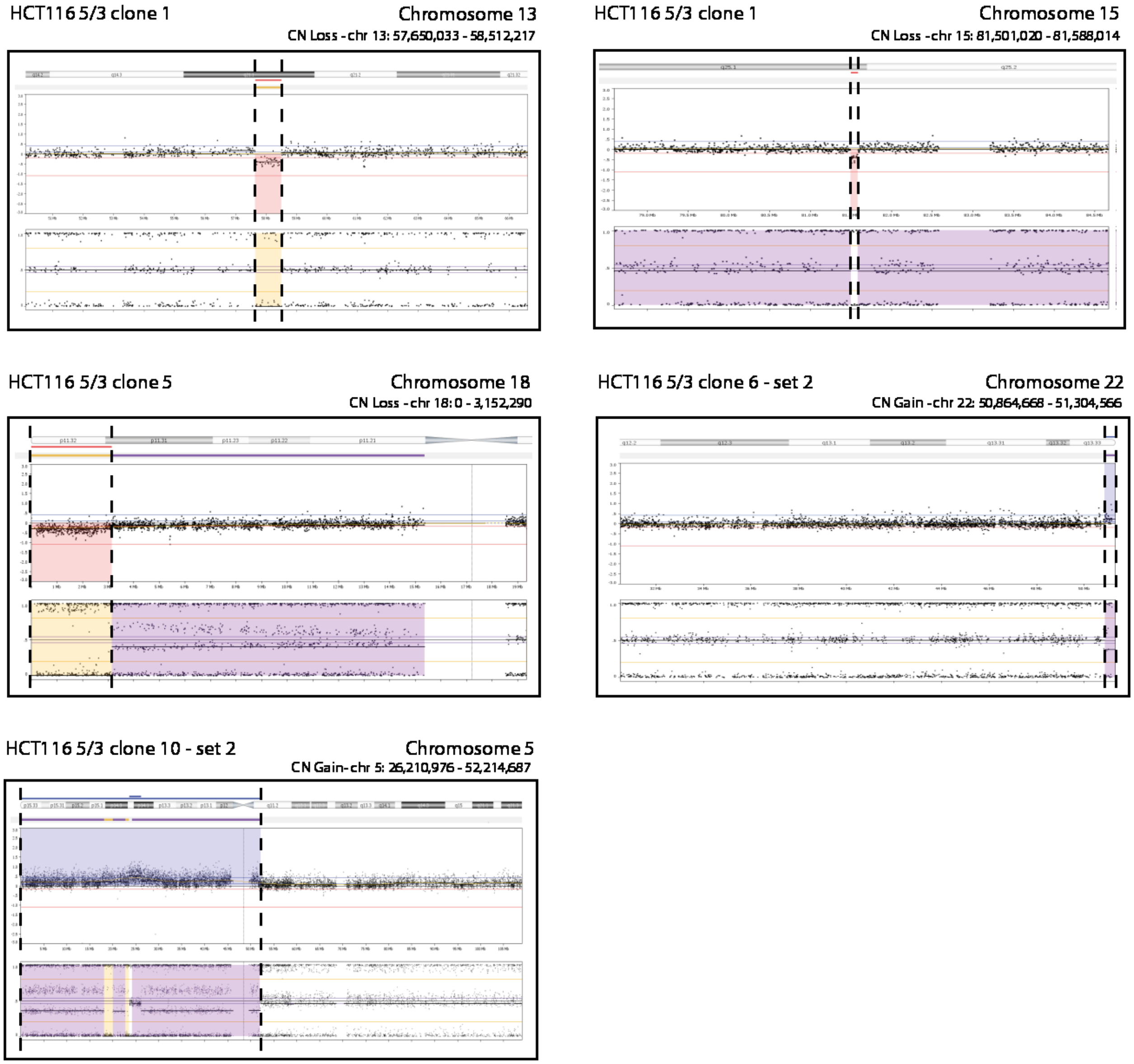

Supplementary Figure 5 Copy number alterations in trisomic cells

Unique *de novo* copy number alterations determinated in the individual clones derived from single aneuploid cell lines. Two independent sets of 12 single-cell clones were analyzed. (**a**) shows the CNAs from set 1; (**b**) set 2. Blue - copy number gain, red - copy number loss. Yellow and magenta denote the alleles A and B, respectively.

a

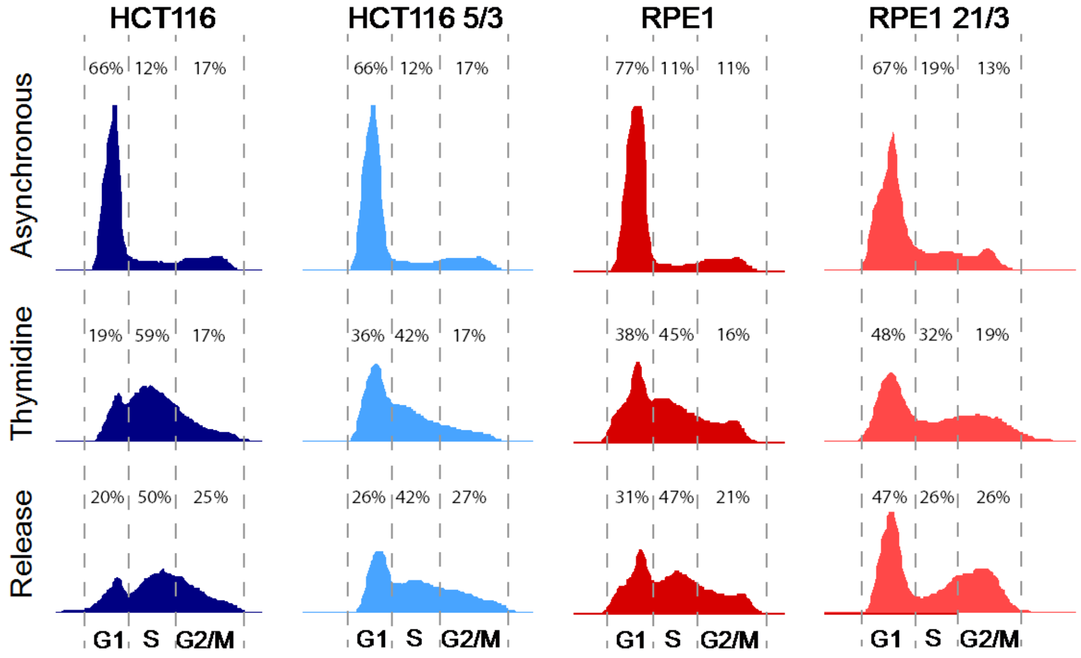

Supplementary Figure 6 Cell cycle profiles after thymidine synchronization and release
(**a**) Cell cycle profile of HCT116, HCT116 5/3, RPE1 and RPE1 21/3 under normal conditions (asynchronous), 30 hours after thymidine addition (thymidine) and 2 hours after thymidine washout (release).

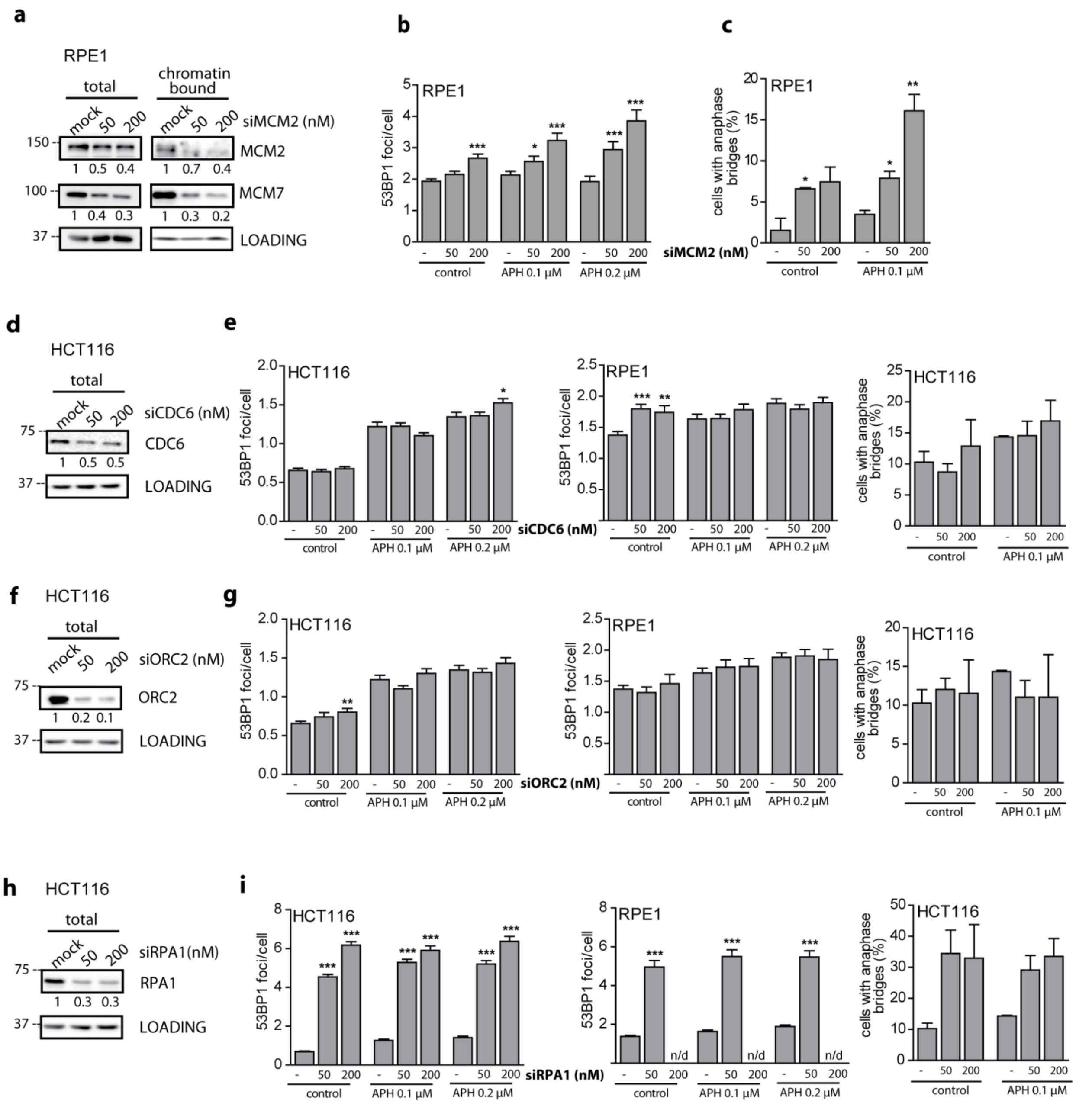

Supplementary Figure 7 Levels of replication proteins in aneuploid cells and the effects of downregulation of replication proteins in control cells

(**a**) Immunoblotting of whole cell lysates and chromatin bound fractions upon partial depletion of MCM2 by siRNA in the parental RPE1 cell line. Note the coordinate decrease in MCM7 abundance. (**b**) Average number of 53BP1 foci and (**c**) average number of anaphase bridges in cells depleted for MCM2. Partial depletion of CDC6 (**d**), ORC2 (**f**) and RPA1 (**h**) by siRNA in the parental HCT116 and RPE1 cell lines. Average number of 53BP1 foci and % of cells with anaphase bridges in cells depleted for CDC6 (**e**), ORC2 (**g**) and RPA1 (**i**). At least two independent experiments were performed and at least 500 cyclin A2-negative or 50 anaphases were scored for 53BP1 foci or anaphase bridge quantification, respectively. All plots show mean ± SEM; non-parametric T-test; * p<=0.05, ** p<=0.01, *** p<=0.001.

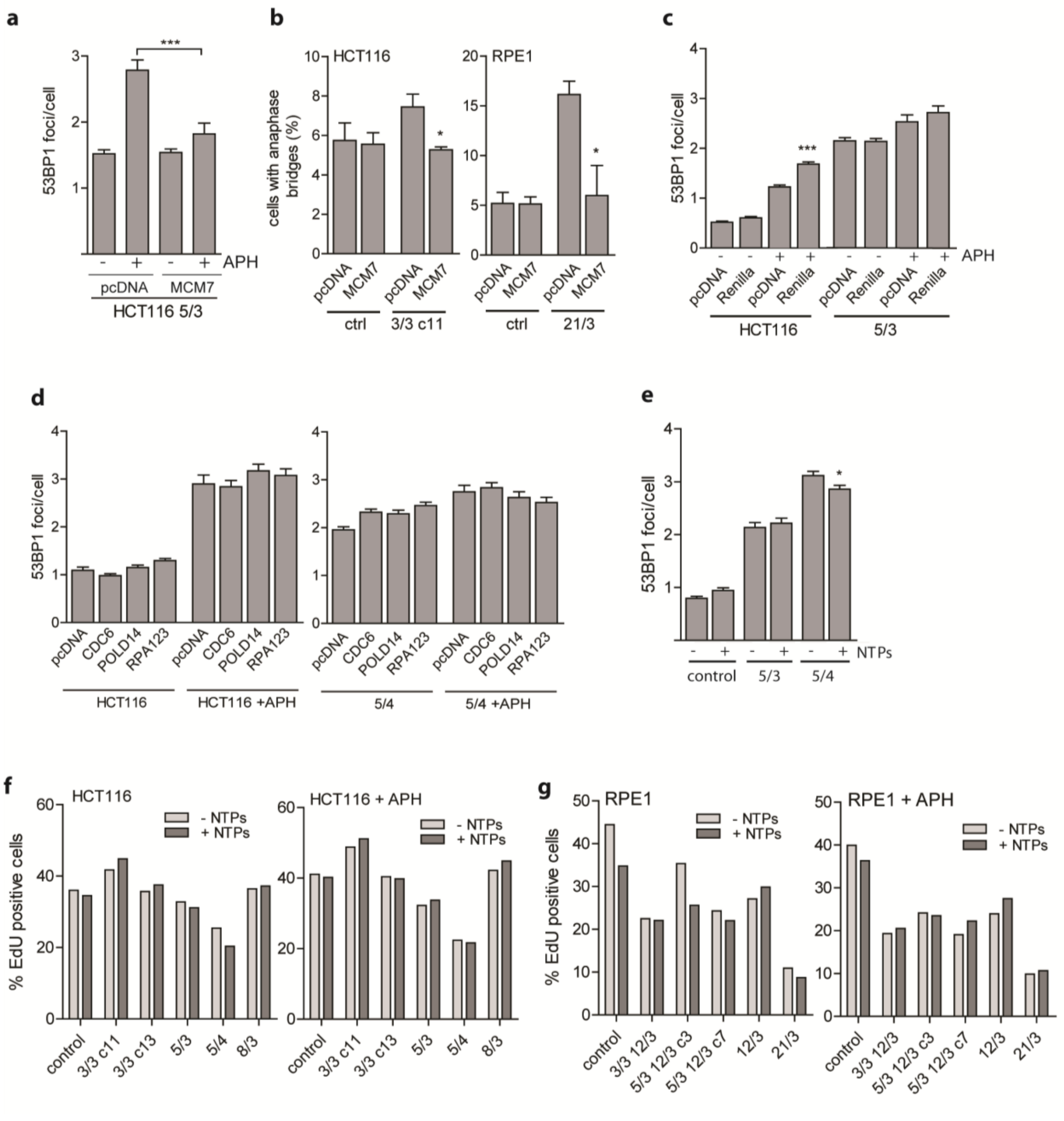

Supplementary Figure 8 The effects of NTP supplementation on EdU incorporation and the occurence of 53BP1 foci in aneuploid cells

(**a**) Accumulation of 53BP1 foci and (b) anaphase bridges upon transient overexpression of MCM7. One representative plot of three independent experiments (a) or mean ± SEM of three independent experiments (**b**) is shown . Non-parametric two-sided T test; *: P<0.05, **: P<0.01, ***: P<0.001.

(**c**) Accumulation of 53BP1 foci in HCT116 and in HCT116 5/3 upon overexpression of Renilla luciferase. (**d**) Accumulation of 53BP1 foci in HCT116 and in HCT116 5/4 upon overexpression of replication factors. (**e**) 53BP1 foci formation in HCT116 and its aneuploid derivatives in the presence or absence of nucleoside supplement. Plot shows the average number of 53BP1 foci of at least 500 cyclin A2-negative cells collected in one experiment. Non-parametric t-test. * p<=0.05% EdU positivity in control HCT116 and aneuploid derivatives (**f**) and RPE1 and aneuploid derivatives (**g**) with or without nucleoside supplement (NTPs).

Fig 3c

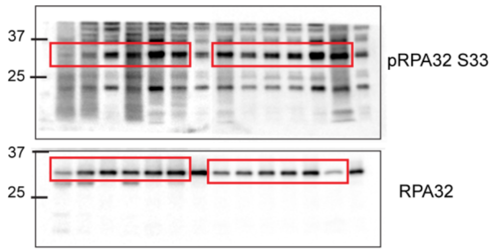

Fig 5b

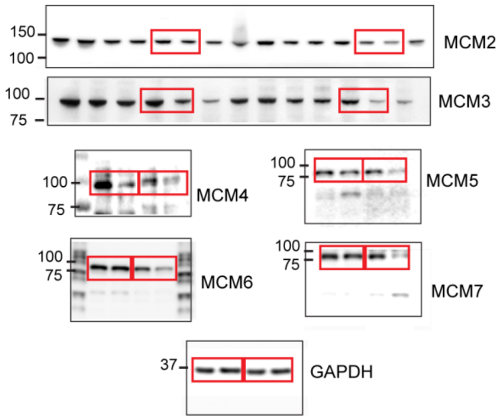

Fig6a

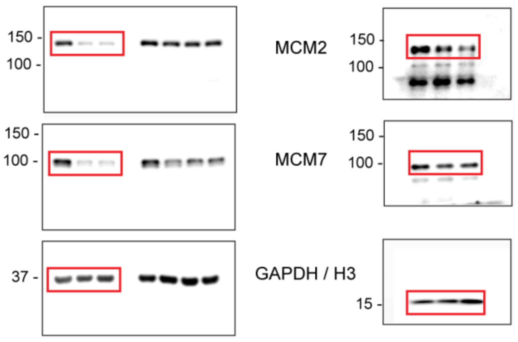

Fig6g

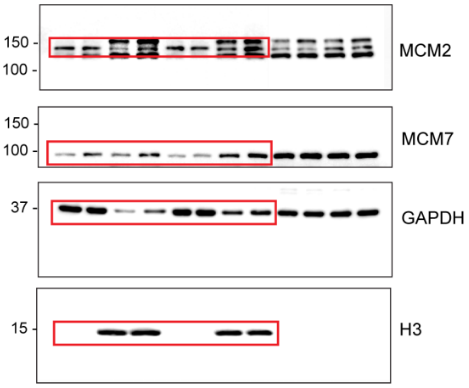

Fig 5c

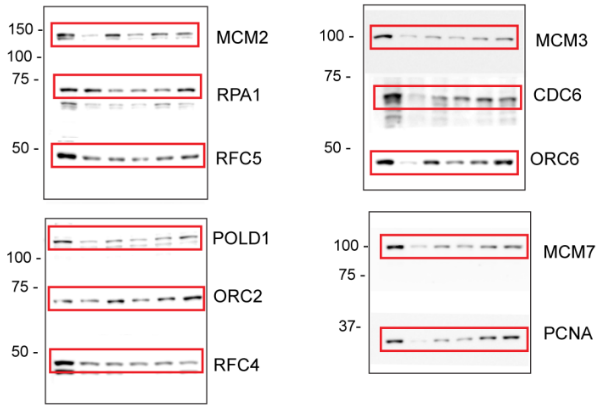

Fig 5d

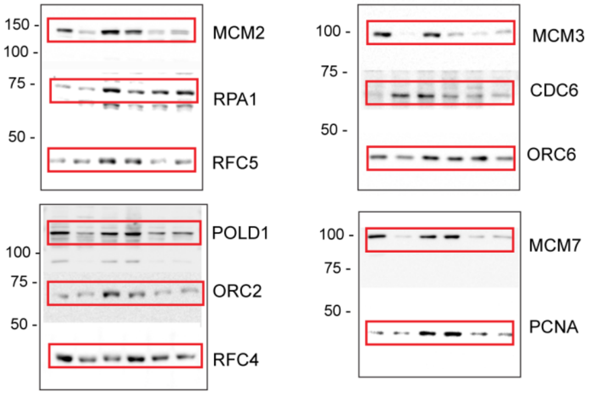

Fig 5e

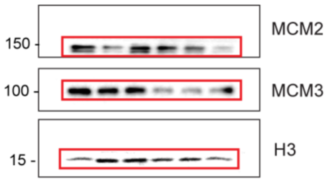

Fig 5f

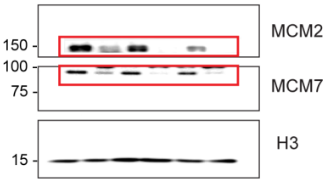

Supplementary Figure 9 Uncropped blots of main figures Frames specify shown bands in indicated main figures.

Fig S4a

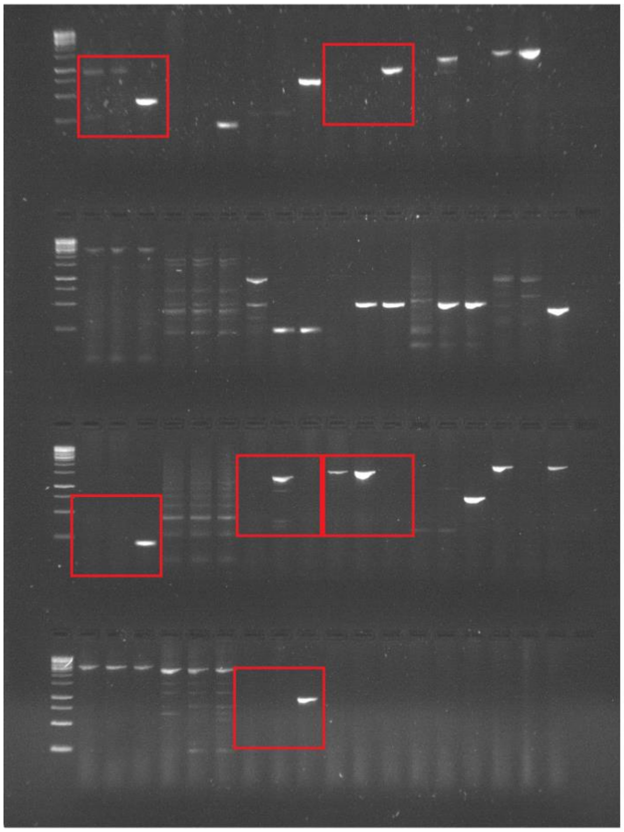

Fig S7a

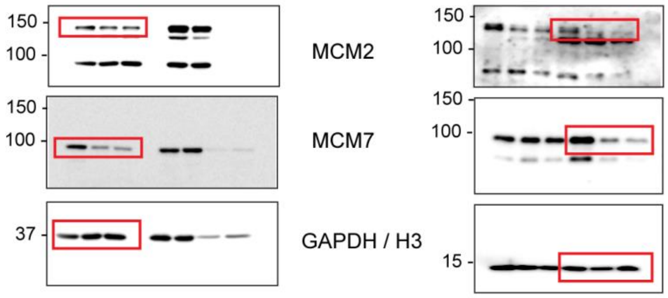

Fig S7d

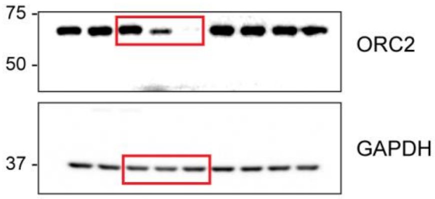

Fig S7f

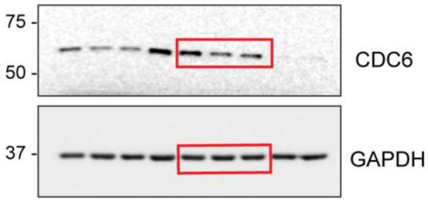

Fig S7g

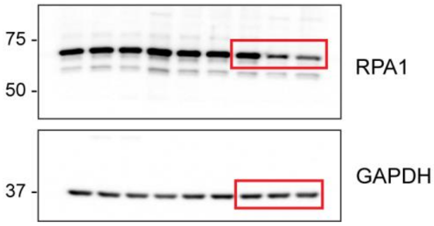

Supplementary Figure 10 Uncropped blots of supplementary figures Frames specify shown bands in indicated supplementary figures.

Supplementary Table 1 List of all cell lines used in the analysis. % of cells with trisomy/tetrasomy was determined by chromosome painting. Note that the cell lines from Koi laboratory were used only for the analysis of the global proteome changes (Figure 5a).

Cell line name used in the text	Origin	Full cell line name	Analysis	% of trisomy or tetrasomy	Remarks
**HCT116**	HCT116 from AATC introduction H2B-GFP	HCT116 H2B-GFP	SNParrays CGH	-	Kuffer et al, 2013
**HCT116 3/3 c11**	MMTC into HCT116 H2B-GFP	HCT116 H2B-GFP 3/3 clone 11	SNParrays CGH	92 %	This work
**HCT116 3/3 c13**	MMTC into HCT116 H2B-GFP	HCT116 H2B-GFP 3/3 clone 13	SNParrays CGH	85 %	This work
**HCT116 5/3**	MMTC into HCT116 H2B-GFP	HCT116 H2B-GFP 5/3 clone 6	SNParrays CGH	92 %	Stingele et al, 2012
**HCT116 5/4**	MMTC into HCT116 H2B-GFP	HCT116 H2B-GFP 5/4 clone 4	SNParrays CGH	83 %	Stingele et al, 2012
**HCT116 8/3**	MMTC into HCT116 H2B-GFP	HCT116 H2B-GFP 8/3 clone 1	SNParrays CGH	78 %	Donnelly et al, 2014
**RPE1**	Taylor laboratory	RPE1 hTERT	SNParrays CGH	-	Kindly provided by Steven Taylor
**RPE1 3/3 12/3**	MMTC into RPE1		SNParrays CGH	100 %	This work Spontaneous gain of chromosome 12
**RPE1 5/3 12/3 c3**	MMTC into RPE1	RPE1 5/3 12/3 clone 3	SNParrays CGH	95 %	Stingele et al, 2012 Spontaneous gain of chromosome 12
**RPE1 5/3 12/3 c7**	MMTC into RPE1	RPE1 5/3 12/3 clone 7	SNParrays CGH	95 %	This work Spontaneous gain of chromosome 12
**RPE1 12/3**	Spontaneously arising trisomy of chromosome 12		CGH	100 %	This work Spontaneous gain of chromosome 12
**RPE1**	Taylor laboratory	RPE1 H2B-GFP hTERT	SNParrays CGH	-	Kindly provided by Steven Taylor
**RPE1 21/3**	MMTC into RPE1 H2B-GFP	RPE1 H2B-GFP 21/3	SNParrays CGH	90 %	Stingele et al, 2012
**HCT116**	Koi laboratory		SNParrays CGH	-	Kindly provided by Minoru Koi Haugen et al, 2008
**HCT116 5/4**	Koi laboratory		SNParrays CGH	88 %	Kindly provided by Minoru Koi Haugen et al, 2008
**HCT116 3/3**	Koi laboratory		CGH	82 %	Kindly provided by Minoru Koi Haugen et al, 2008

Supplementary Table 2 Overview of the identified copy number aberrations in HCT116 5/3.

Sample	chr	start	end	event	Size (bp)	Chr. band	Mosaic	Fragile site overlap
**tr_13_set_2_clone_1**	13	57650033	58512217	CN Loss	862185	q21.1	no	FRA13B
**tr_13_set_2_clone_1**	15	81501020	81588014	CN Loss	86995	q25.1	no	FRA2B/FRA22A
**tr_17_set_2_clone_5**	18	0	3152290	CN Loss	3152291	p11.32 - p11.31	no	
**tr_18_set_2_clone_6**	22	50864668	51304566	CN Gain	439899	q13.33	no	
**tr_22_set_2_clone_10**	5	26210976	52214687	CN Gain	26003712	p14.1 - q11.2	no	FRA5A/FRA5E
**tr_5_set_1_clone_5**	8	142486178	144510494	CN Loss	2024317	q24.3	no	FRA8D
**tr_5_set_1_clone_5**	8	146081828	146364022	CN Loss	282195	q24.3	no	FRA8D
**tr_5_set_1_clone_5**	22	16253076	51304566	CN Gain	35051491	q11.1 - q13.33	no	FRA2A/FRA2B/FRA22A/FRA22B
**tr_6_set_1_clone_6**	4	61577821	61784603	CN Loss	206783	q13.1	no	
**tr_6_set_1_clone_6**	7	155026587	155142658	CN Loss	116072	q36.2 - q36.3	no	FRA7I
**tr_7_set_1_clone_7**	2	172017814	187925525	CN Gain	15907712	q31.1 - q32.1	no	FRA2G/FRA2H
**tr_8_set_1_clone_8**	3	80776725	86007923	CN Loss	5231199	p12.2 - p12.1	no	
